# Repeated Omicron exposures override ancestral SARS-CoV-2 immune imprinting

**DOI:** 10.1038/s41586-023-06753-7

**Published:** 2023-11-22

**Authors:** Ayijiang Yisimayi, Weiliang Song, Jing Wang, Fanchong Jian, Yuanling Yu, Xiaosu Chen, Yanli Xu, Sijie Yang, Xiao Niu, Tianhe Xiao, Jing Wang, Lijuan Zhao, Haiyan Sun, Ran An, Na Zhang, Yao Wang, Peng Wang, Lingling Yu, Zhe Lv, Qingqing Gu, Fei Shao, Ronghua Jin, Zhongyang Shen, Xiaoliang Sunney Xie, Youchun Wang, Yunlong Cao

**Affiliations:** 1 Biomedical Pioneering Innovation Center (BIOPIC), School of Life Sciences, Peking University, Beijing, P. R. China; 2 Changping Laboratory, Beijing, P. R. China; 3 College of Chemistry and Molecular Engineering, Peking University, Beijing, P. R. China; 4 Institute for Immunology, College of Life Sciences, Nankai University, Tianjin, P. R. China; 5 Beijing Ditan Hospital, Capital Medical University, Beijing, P. R. China; 6 Peking–Tsinghua Center for Life Sciences, Tsinghua University, Beijing, P. R. China; 7 Joint Graduate Program of Peking–Tsinghua–NIBS, Academy for Advanced Interdisciplinary Studies, Peking University, Beijing, P. R. China; 8 Sinovac Biotech, Beijing, P. R. China; 9 Organ Transplant Center, NHC Key Laboratory for Critical Care Medicine, Tianjin First Central Hospital, Nankai University, Tianjin, P. R. China; 10 Institute of Medical Biotechnology, Chinese Academy of Medical Science and Peking Union Medical College, Kunming, P. R. China

**Keywords:** Antibodies, Viral infection

## Abstract

The continuing emergence of SARS-CoV-2 variants highlights the need to update COVID-19 vaccine compositions. However, immune imprinting induced by vaccination based on the ancestral (hereafter referred to as WT) strain would compromise the antibody response to Omicron-based boosters^1–5^. Vaccination strategies to counter immune imprinting are critically needed. Here we investigated the degree and dynamics of immune imprinting in mouse models and human cohorts, especially focusing on the role of repeated Omicron stimulation. In mice, the efficacy of single Omicron boosting is heavily limited when using variants that are antigenically distinct from WT—such as the XBB variant—and this concerning situation could be mitigated by a second Omicron booster. Similarly, in humans, repeated Omicron infections could alleviate WT vaccination-induced immune imprinting and generate broad neutralization responses in both plasma and nasal mucosa. Notably, deep mutational scanning-based epitope characterization of 781 receptor-binding domain (RBD)-targeting monoclonal antibodies isolated from repeated Omicron infection revealed that double Omicron exposure could induce a large proportion of matured Omicron-specific antibodies that have distinct RBD epitopes to WT-induced antibodies. Consequently, immune imprinting was largely mitigated, and the bias towards non-neutralizing epitopes observed in single Omicron exposures was restored. On the basis of the deep mutational scanning profiles, we identified evolution hotspots of XBB.1.5 RBD and demonstrated that these mutations could further boost the immune-evasion capability of XBB.1.5 while maintaining high ACE2-binding affinity. Our findings suggest that the WT component should be abandoned when updating COVID-19 vaccines, and individuals without prior Omicron exposure should receive two updated vaccine boosters.

## Abstract (web summary)

Exposure to early variants of SARS-CoV-2 results in immune imprinting in mouse models and in humans, reducing neutralizing antibody titres against Omicron variants, which could be mitigated with multiple updated boosters.

## Main

SARS-CoV-2 continues to evolve, and new mutants emerge under continuous humoral immune pressure^6–14^. New variants, such as the XBB lineages, are capable of evading antibodies induced by vaccination or infection, resulting in repeated infections among populations^1,3,15,16^. Therefore, it is critical to develop updated vaccines that can elicit strong immune responses against the latest variants.

mRNA vaccine platforms can quickly adapt to new SARS-CoV-2 variants^17–20^. However, as the majority of the population was vaccinated with the ancestral SARS-CoV-2 strain (WT), immune imprinting induced by WT vaccination presents a major challenge to the performance of updated boosters^21,22^. This is because boosting with a variant that is antigenically distinct from WT would mostly recall memory B cells induced by WT vaccination and mask the de novo generation of variant-specific B cells, which would hinder the generation of appropriate humoral immunity against new and emerging variants^2,3,5,23–27^.

It is crucial to explore vaccination strategies that can counter immune imprinting. In this paper, we investigated the dynamics of immune imprinting in both mouse models and human cohorts, with a particular focus on whether repeated exposure to Omicron variants could alleviate immune imprinting.

## Alleviation of immune imprinting in mice

First, we investigated the effects of SARS-CoV-2 immune imprinting induced by WT vaccination in BALB/c mice. To accomplish this, two doses of 3 μg CoronaVac (an inactivated vaccine derived from WT SARS-CoV-2) were used as primary immunization, and variant spike proteins were used as boosters^28–30^. All SARS-CoV-2 spike proteins contained six proline substitutions (S6P) and alanine substitutions in the furin cleavage site to stabilize them in the prefusion conformation^31^.

**Fig. 1:**
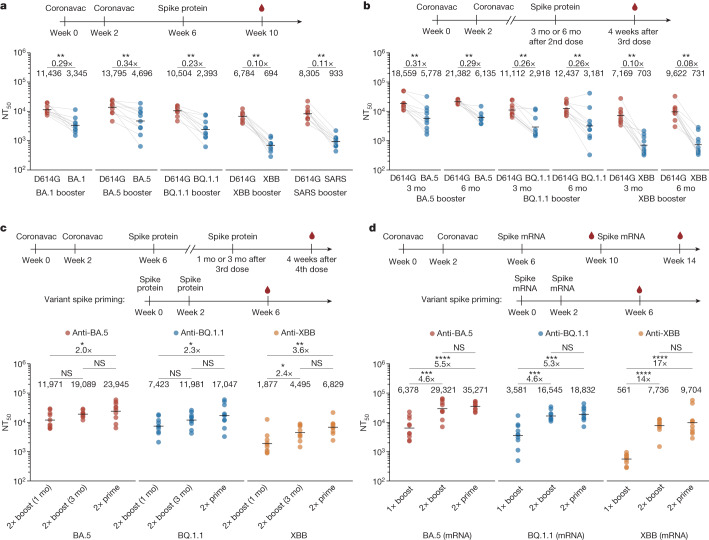
Humoral immune imprinting in mice. **a**, NAb response after two doses of priming with CoronaVac followed by boosting with SARS-CoV-1 spike protein or SARS-CoV-2 variant spike proteins in mice. **b**, NAb response after 2 doses of CoronaVac priming followed by boosting with variant spike proteins with 3-month (mo) or 6-month time intervals in mice. **a**,**b**, The *x*-axis labels indicate NT_50_ values against the respective variants and the variants used for boosting are indicated at the bottom of the figure; fold differences in titres against variants compared with D614G are shown above the line. **c**, NAb response after priming with 2 doses of variant spike proteins or priming with 2 doses of CoronaVac followed by 2 boosts of variant spike proteins with 1-month or 3-month intervals in mice. **d**, NAb response after priming with two doses of variant spike mRNAs or priming with two doses of CoronaVac followed by two boosts of variant spike mRNAs. **c**,**d**, The variants used for priming or boosting are indicated at the bottom of the figure and red, blue, yellow circles indicate NT_50_ values for BA.5, BQ.1.1 and XBB. Ten mice were immunized and analysed in each group (*n* = 10) except in **b** eight mice were immunized with BA.5 booster 6 months after priming (*n* = 8). The dosage of CoronaVac, spike protein and spike mRNA were 3 μg, 10 μg and 1 μg, respectively. Sera were collected four weeks after the last dose. Geometric mean titres (GMTs) are shown. Two-tailed Wilcoxon signed-rank tests for paired samples in **a**,**b** and two-tailed Wilcoxon rank-sum tests for independent samples in **c**,**d**. *P < 0.05, **P < 0.01, ***P < 0.001, ****P < 0.0001; NS, not significant (*P* > 0.05). All neutralization assays were conducted as at least two independent experiments. Source Data

**Extended Data Fig. 1:**
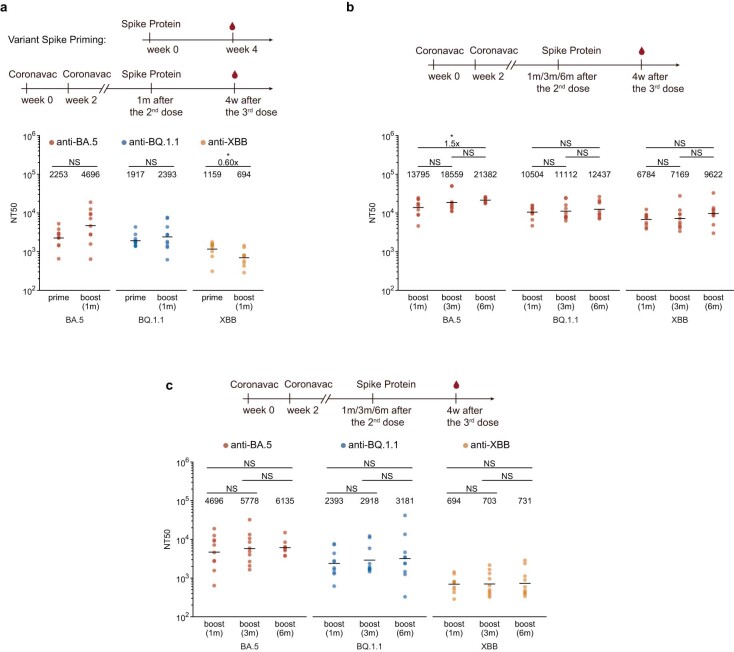
Neutralizing antibody response after CoronaVac priming and one-dose variant spike boosting. **a**, Comparison of neutralizing titers between mice immunized with one doses of BA.5/BQ.1.1/XBB Spike protein and mice with 2 doses of CoronaVac followed by one-dose Spike protein boosters. The variants used for priming or boosting are indicated at the bottom of the figure and red, blue, yellow circles indicate the NT50s against BA.5, BQ.1.1, and XBB. **b**, **c**, Comparison of neutralizing titers among different groups of mice immunized with 2 doses of CoronaVac followed by one-dose BA.5/BQ.1.1/XBB Spike protein boosters administered with one-month, three-month, or six-month intervals between the second and third dose. **b**) Neutralizing titers against D614G; **c**) Neutralizing titers against variants that the mice boosted with. The variants used for priming or boosting are indicated at the bottom of the figure and red, blue, yellow circles indicate the NT50s against BA.5, BQ.1.1, and XBB. 10 mice were immunized and analyzed in each group (n = 10), except in **b,c** eight mice were immunized with BA.5 booster 6 months after priming (n = 8), and all neutralization assays were conducted in at least two independent experiments. Sera were collected four weeks after the last dose. Geometric mean titers (GMT) were labeled. All neutralization assays were conducted in at least two independent experiments. Statistical significance was determined using the two-tailed Wilcoxon rank sum test. *p < 0.05, **p < 0.01, ***p < 0.001, ****p < 0.0001, and not significant (NS) p > 0.05. Source Data

Mice that received a single booster of 10 μg spike protein, including BA.1, BA.5, BQ.1.1, XBB and SARS-CoV-1, exhibited lower serum 50% neutralizing titre (NT_50_) values against D614G (using vesicular stomatitis virus (VSV)-based pseudovirus) as the antigenic distance between the boosting variant and WT increased, suggesting decreased cross-reactive B cell recall after the variant booster (Fig. 1a). Additionally, single-dose boosted mice had significantly lower NT_50_ against the boosting variants compared to D614G (Fig. 1a). Moreover, single-dose boosting with XBB spike generated lower NT_50_ values against XBB lineages than those observed in the one-dose XBB priming group (Extended Data Fig. 1a). These results revealed substantial ancestral strain immune imprinting at the serum level, and are consistent with the observations in humans^2,3,23,24,32,33^, as well as previous findings of immune imprinting in influenza viruses^34,35^.

To investigate whether prolonging the interval between the primary WT immunization and the variant booster could alleviate immune imprinting, we further tested boosting mice three and six months after CoronaVac priming (Fig. 1b). The 3-month and 6-month intervals between WT priming and variant boosting slightly increased overall NT_50_ values, but the fold difference between NT_50_ values against D614G and XBB spike remained high (Fig. 1b). Also, there was no significant difference in NT_50_ among 1-month, 3-month and 6-month boosting interval groups for BQ.1.1 and XBB boosting (Extended Data Fig. 1b,c). This suggests that longer intervals between priming and Omicron boosting—which would allow the maturation of WT-induced antibodies—may not be sufficient to alleviate immune imprinting.

**Extended Data Fig. 2:**
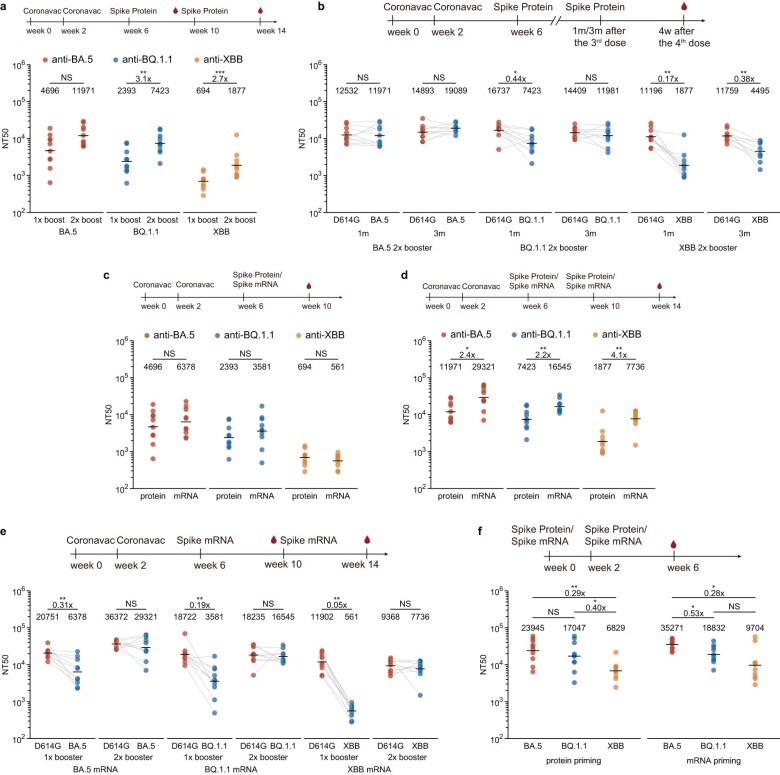
Neutralizing antibody response after CoronaVac priming and two-dose variant spike booster or two-dose variant spike priming. **a**, Comparison of neutralizing titers after CoronaVac priming and one-dose or two-dose variant spike boosting. **b**, D614G and boosting variant neutralizing titers after CoronaVac priming and two-dose variant spike boosting. **c**-**d**, Comparison of neutralizing titers after CoronaVac priming and variant spike protein or mRNA boosting. one-dose boosting in **c** and two-dose boosting in **d**. **e**, Neutralizing antibody titers after CoronaVac priming and one-dose or two dose variant spike mRNA boosters. **f**, Neutralizing antibody titers after two-dose variant spike mRNA or protein boosters. 10 mice were immunized and analyzed in each group (n = 10), and all neutralization assays were conducted in at least two independent experiments. Sera were collected four weeks after the last dose. Geometric mean titers (GMT) were labeled. All neutralization assays were conducted in at least two independent experiments. Statistical significance was determined using the two-tailed Wilcoxon rank sum test (a, c, d and f) or two-tailed Wilcoxon signed-rank test (b and e). *p < 0.05, **p < 0.01, ***p < 0.001, ****p < 0.0001, and not significant (NS) p > 0.05. Source Data

Next, we examined how second Omicron boosters perform^36^. We started by boosting CoronaVac-primed mice with two doses of the variant spike protein over a 1-month or 3-month interval (Fig. 1c). Notably, the second boosters resulted in greatly increased NT_50_ values against the corresponding variants (Extended Data Fig. 2a), as well as substantially reduced fold differences between D614G and variant spike proteins (Extended Data Fig. 2b). However, the neutralizing titres induced by two boosters over a 1-month interval after two doses of CoronaVac priming were still lower than those induced by 2 doses of variant priming, clearly indicating the interference caused by immune imprinting (Fig. 1c). Notably, compared with a 1-month boosting interval, a 3-month interval between Omicron boosters resulted in clear improvements in NT_50_ values against all the corresponding boosting variants (Fig. 1c), and the fold difference between the NT_50_ against D614G and the boosting variants also decreased (Extended Data Fig. 2b). This indicates that the maturation of B cells induced by Omicron boosting are highly beneficial for mitigation of immune imprinting.

As mRNA vaccines encoding spike have proved to be capable of quick adaptation to new variants, it is critical to test how updated mRNA variant boosters perform, especially when the higher immunogenicity of mRNA vaccine might help to alleviate immune imprinting when served as Omicron boosters. Therefore, we tested 1 μg mRNA vaccines encoding BA.5, BQ.1.1 and XBB spike as boosters, replacing the protein boosters (Fig. 1d). As expected, 1 μg mRNA vaccine demonstrated higher immunogenicity than the 10 μg spike protein vaccine (Extended Data Fig. 2c,d,f). However, the performance of one-dose mRNA Omicron boosters was still affected by immune imprinting, whereas two mRNA Omicron boosters could significantly increase antibody titres and achieve similar titres compared with the priming groups (Fig. 1d and Extended Data Fig. 2c–e). This suggests that increasing the immunogenicity of variant boosters could help to counter immune imprinting from WT vaccination.

Notably, among the Omicron variants tested, XBB boosting exhibited the lowest overall titres (Fig. 1c,d). Indeed, these variant vaccines, whether they used protein or mRNA, exhibited different levels of immunogenicity in mice, with XBB having the lowest immunogenicity (Extended Data Fig. 2f).

Together, our results observed in mice emphasize that the efficacy of the first Omicron booster is severely limited, and a second booster is almost mandatory to alleviate immune imprinting and generate high antibody responses, especially for boosters encoding variants that exhibit long antigenic distance from WT, such as XBB.

## Mitigating immune imprinting in humans

**Fig. 2:**
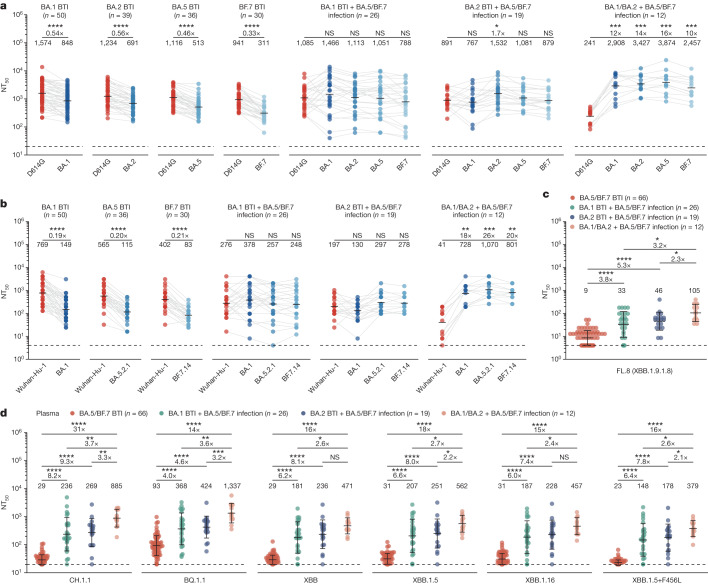
Humoral immune imprinting after repeated Omicron infections in humans. **a**, Examination of immune imprinting after Omicron breakthrough infections and repeated infections. Plasma antibody titres against pseudotyped D614G and variants were measured. **b**, Plasma antibody titres against authentic variants. **a**,**b**, Fold changes in titres against variants compared with D614G or Wuhan-Hu-1 are displayed above the line, GMTs are shown; two-tailed Wilcoxon signed-rank test. **c**, Plasma antibody titres against authentic FL.8 (XBB.1.9.1.8). **d**, Plasma antibody breadth after one-time breakthrough infection and repeated Omicron infections. Plasma antibody titres against circulating pseudotyped variants were measured. **c**,**d**, Fold changes in titres between different cohorts are shown above the line; two-tailed Wilcoxon rank-sum tests. BA.1, BA.2, BA.5 and BF.7 BTI: post-vaccination BA.1, BA.2, BA.5 or BF.7 breakthrough infection. BA.1, BA.2 BTI + BA.5/BF.7 infection: post-vaccination either BA.1 or BA.2 breakthrough infection followed by BA.5/BF.7 reinfection. BA.1/BA.2 + BA.5/BF.7 infection: either BA.1 or BA.2 infection followed by BA.5/BF.7 reinfection with no SARS-CoV-2 vaccination history. BA.1 BTI, *n* = 50; BA.2 BTI, *n* = 39; BA.5 BTI, *n* = 36; BF.7 BTI, *n* = 30; BA.1 BTI + BA.5/BF.7 infection, *n* = 26; BA.2 BTI + BA.5/BF.7 infection, *n* = 19; BA.1/BA.2 + BA.5/BF.7 infection, *n* = 12. *n* refers to the number of individuals. Blood samples were collected 1–2 months after the last infection. Detailed information about the cohorts is presented in Supplementary Table 1. **c**,**d**, Data are GMT ± s.d. Dashed lines indicate the limit of detection (NT_50_ = 20 and NT_50_ = 4 for pseudovirus and authentic virus neutralization assays, respectively). All neutralization assays were conducted as at least two independent experiments.

**Extended Data Fig. 3:**
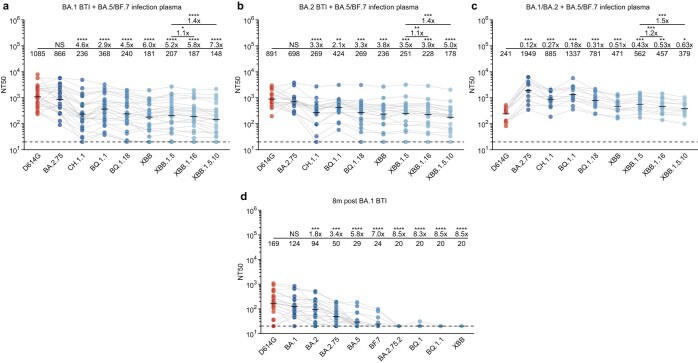
Antibody breadth of plasma after repeated Omicron infections. **a**-**d**, Plasma antibody titers against pseudotyped D614G and variants after (**a**) BA.1 BTI + BA.5/BF.7 infection (n = 26), (**b**) BA.2 BTI + BA.5/BF.7 infection (n = 19), (**c**) BA.1/BA.2 + BA.5/BF.7 infection (n = 12), **d**) 8 month post BA.1 BTI (n = 22). ‘n’ refers to the number of individuals. Fold changes between titers against variants and D614G were calculated and shown above the line. Statistical significance was determined using the two-tailed Wilcoxon signed-rank test. *p < 0.05, **p < 0.01, ***p < 0.001, ****p < 0.0001, and not significant (NS) p > 0.05.

**Extended Data Fig. 4:**
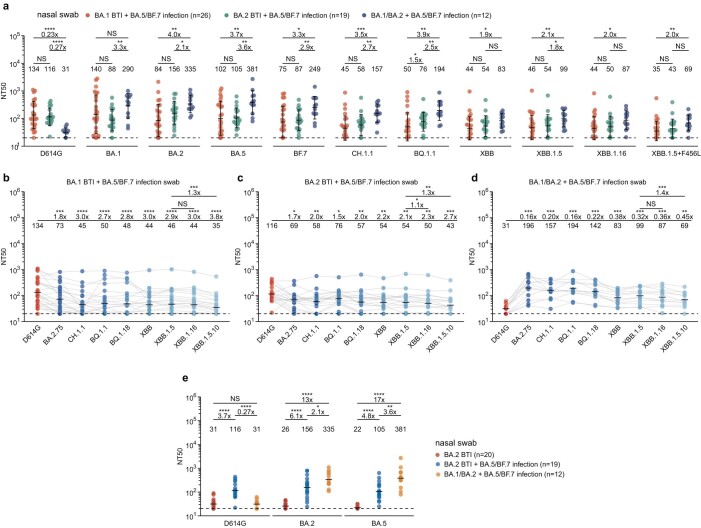
Neutralizing titers of nasal swabs after repeated Omicron infections. **a**, Comparison of nasal swab neutralizing titers among repeated Omicron infection cohorts. Nasal swab antibody titers against pseudotyped variants were measured. Fold changes between titers of different cohorts were calculated and shown above the line. Statistical significance was determined using the two-tailed Wilcoxon rank sum test. Geometric mean ± SD are labeled. **b**-**d**, Nasal swab antibody titers against pseudotyped D614G and variants after (**b**) BA.1 BTI + BA.5/BF.7 infection (n = 26), (**c**) BA.2 BTI + BA.5/BF.7 infection (n = 19), (**d**) BA.1/BA.2 + BA.5 infection (n = 12). n’ refers to the number of individuals. Fold changes between titers against variants and D614G were calculated and shown above the line. Statistical significance was determined using the two-tailed Wilcoxon signed-rank test in (b-d). **e**, Comparison of nasal swab antibody titers against pseudotyped D614G and variants among one-time breakthrough infection and repeated infection cohorts. Statistical significance was determined using the two-tailed Wilcoxon rank sum test in (**e**). *p < 0.05, **p < 0.01, ***p < 0.001, ****p < 0.0001, and not significant (NS) p > 0.05.

To verify whether the findings obtained from mice also apply to humans, we recruited cohorts with repeated Omicron breakthrough infections (BTIs), including individuals with post-vaccination either BA.1 or BA.2 BTI followed by BA.5/BF.7 (the specific variant was not determined) reinfection (BTI + reinfection) and compared them to previously reported BA.1, BA.2, BA.5, BF.7 one-time BTI cohorts^3,32,37,38^. Of note, we also included individuals who had no history of SARS-CoV-2 vaccination before repeated infection (vaccination-naive reinfection) as controls (Supplementary Table 1). We first examined plasma neutralizing titres against exposed variants using neutralizing assays with pseudovirus and authentic virus (Fig. 2a,b). Similar to the results for mice immunization, human plasma neutralizing titres induced by one-time Omicron BTIs against the corresponding variant were significantly lower than those against D614G, consistent with our previous report^3^, and the fold difference between the NT_50_ values for D614G and those against corresponding variants also increased as the antigenic distance increased (Fig. 2a,b). As expected, in the repeated Omicron infection group—with or without SARS-CoV-2 vaccination history—the neutralizing titres against Omicron variants significantly increased compared with one-time BTIs (Fig. 2a,b). Crucially, BA.1 or BA.2 BTI followed by BA.5/BF.7 reinfections demonstrated similar NT_50_ values between exposed Omicron variants and D614G, indicating alleviation of immune imprinting by the second Omicron exposure (Fig. 2a,b). However, the NT_50_ values for the vaccination-naive reinfection group against Omicron variants were the highest among these cohorts (Fig. 2a,b), suggesting that repeated BTIs were still prone to WT vaccination-induced immune imprinting. Compared with one-time BTIs, repeated Omicron infection also led to an increase in the neutralizing titres against highly immune-evasive CH.1.1, BQ.1.1, XBB, FL.8 (XBB.1.9.1.8), XBB.1.5, XBB.1.16 and XBB.1.5 + F456L variants (Fig. 2c,d and Extended Data Fig. 3a–c), indicating that repeated Omicron infections may broaden the breadth of the antibody response. In addition, we found that the nasal swab samples from individuals with repeated Omicron infection exhibited higher neutralizing titres against Omicron variants than one-time breakthrough infection, suggesting that strong nasal mucosal humoral immunity had been established after repeated infection (Extended Data Fig. 4).

**Fig. 3:**
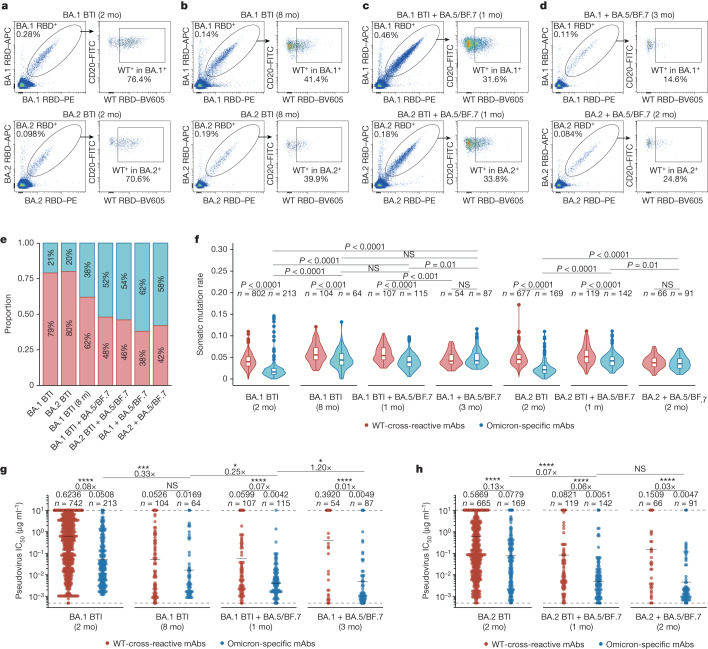
B cell immune imprinting after repeated Omicron infections. **a**–**d**, Flow cytometry analysis of pooled B cells from individuals who had recovered from Omicron infection. BA.1 (top) and BA.2 (bottom) RBD double-positive CD20^+^IgM^−^IgD^−^CD27^+^ B cells were isolated for paired-single-cell V(D)J sequencing. Flow cytometry analyses were performed in cohorts of the following: 2 months after BA.1 (top) or BA.2 (bottom) breakthrough infections (**a**), 8 months after BA.1 (top) or BA.2 (bottom) breakthrough infections (**b**), 1 month after BA.5/BF.7 reinfection after BA.1 (top) and BA.2 (bottom) breakthrough infections (**c**), and 2–3 months after BA.5/BF.7 reinfection after BA.1 (top) or BA.2 (bottom) infection without SARS-CoV-2 vaccination history (**d**). APC, allophycocyanin; FITC, fluorescein isothiocyanate; PE, phycoerythrin; BV605, Brilliant Violet 605. **e**, Proportions of WT-binding and non-WT-binding antibodies from Omicron breakthrough infection and repeated Omicron infection cohorts. Binding specificity was determined by ELISA. The antibodies were expressed in vitro using the sequence of the RBD-binding memory B cells from various cohorts. **f**, The heavy-chain variable domain somatic hypermutation rate of the monoclonal antibodies (mAbs) from various cohorts. Two-tailed Wilcoxon rank-sum tests. Boxes indicate the 25th percentile, median and 75th percentile, and whiskers extend to median ± 1.5 times the interquartile range. Violin plots show kernel density estimation curves of the distribution. The numbers and ratios of samples in each group are labelled above the violin plots. **g**,**h**, The BA.1 (**g**) or BA.2 (**h**) pseudovirus-neutralizing ability (IC_50_) of monoclonal antibodies from various cohorts. Detection limit is denoted as a dashed line, and geometric mean is denoted as black bar. Geometric mean, fold changes and the number of antibodies are indicated above the plots. **f**–**h**, Two-tailed Wilcoxon rank-sum tests.

Neutralization data from both mouse and human studies underscore the crucial role of secondary Omicron exposure in mitigating immune imprinting and generating broad antibody responses. We propose that this is largely attributable to the further expansion of Omicron-specific memory B cells generated de novo by the first Omicron exposure. To assess whether this is the case, we first analysed the Omicron specificity of RBD-binding memory B cells from BTIs, BTIs + reinfection, and vaccine-naive reinfection cohorts using fluorescence-activated cell sorting (FACS) (Supplementary Data 1). As we previously reported, in one-time Omicron BTI cohorts, more than 70% of the Omicron RBD-binding memory B cells also bound to WT RBD, indicating that post-vaccination Omicron infection mainly recalls cross-reactive memory B cells elicited by WT-based vaccination (Fig. 3a). Subsequently, following an extended duration of time (eight months) after the first Omicron BTI, the proportion of cross-reactive cells declined, whereas that of Omicron-specific cells increased, suggesting that longer B cell maturation periods increased the proportion of Omicron-specific memory B cells (Fig. 3b). Nevertheless, at eight months after BA.1 BTI, the plasma neutralizing titres were very low owing to antibody waning, and thus required a secondary Omicron boosting to increase the antibody levels (Extended Data Fig. 3d). Notably, for Omicron BTI + reinfection cohorts, the proportion of cross-reactive cells declined further but still remained higher than that observed in the vaccination-naive reinfection cohort (Fig. 3c,d). These results are highly correlated with the plasma NT_50_ values of the cohorts, which suggests that Omicron-specific antibodies are a major contributor to the increased antibody breadth and neutralization capability after repeated Omicron infection.

To further investigate the potency, breadth and epitopes of these antibodies, the BA.1 RBD-binding cells and BA.2 RBD-binding cells from the various BA.1/BA.2 infection cohorts were sorted and sequenced by high-throughput single-cell V(D)J sequencing. Antibodies were then expressed in vitro as human IgG1 monoclonal antibodies (Supplementary Table 2). For one-time Omicron BTI cohorts, enzyme-linked immunosorbent assay (ELISA) confirmed that approximately 20% of the isolated monoclonal antibodies specifically bound to the BA.1/BA.2 RBD and were not cross-reactive to WT RBD, which was consistent with FACS results (Fig. 3e). Furthermore, long-term sampling (eight months) after BA.1 BTI yielded an increased proportion of BA.1 RBD-specific monoclonal antibodies compared with short-term (two months after BA.1 BTI) sampling. Moreover, reinfection with BA.5/BF.7 further increased the proportion of BA.1 or BA.2 RBD-specific monoclonal antibodies to around 50%, but this was still lower than that in vaccination-naive reinfection groups (Fig. 3e). Notably, the somatic hypermutation (SHM) rates of BA.1/BA.2-specific antibodies in BTI + reinfection cohorts were higher than that in one-time BTI cohorts (Fig. 3f), and the increased affinity maturation of BA.1/BA.2-specific antibodies contributes to their increased potency against Omicron variants (Fig. 3g,h). Together, these data indicate that long-term maturation after one-time Omicron BTI and repeated Omicron infections could significantly raise the proportion and maturation of Omicron-specific antibodies, greatly contributing to the increased plasma neutralization potency against Omicron variants.

## Epitope analyses of Omicron-specific antibodies

To further interrogate the composition of antibodies elicited by Omicron BA.5/BF.7 BTI and reinfection, we determined the binding sites and escaping mutations on RBD of these monoclonal antibodies using deep mutational scanning^39,40^ (DMS). As the proportion of Omicron-specific antibodies is indispensable in reinfection cohorts, and the last exposure of all cohorts involved in this study is BA.5/BF.7, we built a yeast display mutant library based on the BA.5 RBD and performed DMS for these monoclonal antibodies in a high-throughput manner, similar to previously described WT-based methods^40^. To enhance the sampling of Omicron-specific neutralizing antibodies (NAbs) to facilitate epitope characterization, we specifically isolated an additional panel of RBD-targeting monoclonal antibodies that did not cross-bind to WT according to feature barcode counting during the paired-single-cell V(D)J sequencing and determined their BA.5-based DMS data. We also determined the BA.5-based DMS data for all BA.5-RBD-binding monoclonal antibodies from previous collections isolated from various immune backgrounds (Supplementary Table 2). In total, a comprehensive panel consisting of BA.5-based DMS for 1,350 monoclonal antibodies was collected.

**Fig. 4:**
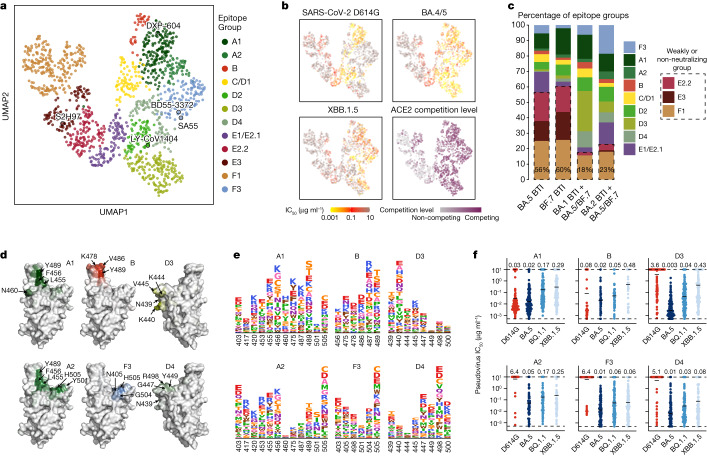
Epitope distribution and characterization of monoclonal antibodies elicited by Omicron BTI and reinfection. **a**, UMAP embedding of epitope groups of monoclonal antibodies binding BA.5 RBD isolated from convalescent individuals who experienced BA.5/BF.7 BTI or reinfection (*n* = 1,350). **b**, Neutralization activities, denoted as IC_50_ values, for SARS-CoV-2 D614G (*n* = 1,349), BA.4/5 (*n* = 1,322) and XBB.1.5 (*n* = 1,346) spike-pseudotyped VSV, and ACE2 competition determined by ELISA (*n* = 1,344), are projected onto the UMAP embedding space. **c**, Distribution of monoclonal antibodies across epitope groups is shown for BA.5 BTI, BF.7 BTI, BA.1 BTI with BA.5/BF.7 reinfection and BA.2 BTI with BA.5/BF.7 reinfection. Epitope groups predominantly comprising non-neutralizing or weakly neutralizing monoclonal antibodies (E2.2, E3 and F1) are highlighted with dashed boxes. The percentage of antibodies in these three groups is labelled on each bar. **d**, Average DMS escape scores of the crucial epitope groups contributing to neutralization against XBB.1.5 are indicated on the structure model of the SARS-CoV-2 BA.5 RBD (PDB: 7XNS). Key residues with high escape scores for each group are labelled. **e**, The average DMS escape scores for the key epitope groups are represented as sequence logos; residues are depicted using the standard one-letter code and coloured on the basis of their chemical properties. The height of each letter corresponds to the escape score of the respective mutation. **f**, Pseudovirus-neutralization activities of monoclonal antibodies in the six crucial epitope groups (A1 (*n* = 170), A2 (*n* = 60), B (*n* = 33), F3 (*n* = 129), D3 (*n* = 155) and D4 (*n* = 80); *n* refers to the number of monoclonal antibodies) are shown against SARS-CoV-2 D614G, BA.5, BQ.1.1 and XBB.1.5. Geometric mean IC_50_ values are displayed as bars and indicated above each group of data points.

**Extended Data Fig. 5:**
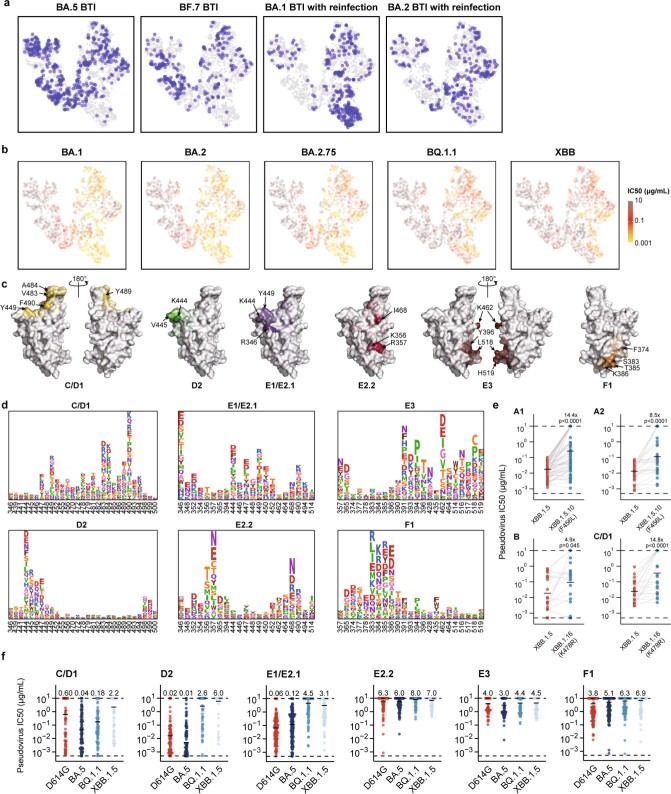
Characteristics of BA.5-reactive mAbs elicited by BA.5/BF.7 BTI or reinfection. **a**, Source of the antibodies are projected onto the UMAP embedding space. Antibodies from BA.5 BTI (n = 445), BF.7 BTI (n = 243), BA.1 BTI with reinfection (n = 284), and BA.2 BTI with reinfection (n = 232) are colored blue in the corresponding panel, and other antibodies are gray. **b**, Neutralization activities, denoted as IC50 values, against SARS-CoV-2 BA.1 (n = 1260), BA.2 (n = 1238), BA.2.75 (n = 1238), BQ.1.1 (n = 1335) and XBB (n = 1341) spike-pseudotyped VSV are projected onto the UMAP embedding space. **c**, Average escape scores of epitope groups that are not shown in Fig. 4d (C/D1, D2, E1/E2.1, E2.2, E3, and F1) are illustrated on the structure model of the SARS-CoV-2 BA.5 RBD (PDB: 7XNS). Key residues with high escape scores for each group are labeled. **d**, Average DMS escape scores for these epitope groups are represented as sequence logos; residues are depicted using the standard one-letter code and colored based on their chemical properties. The height of each letter corresponds to the escape score of the respective mutation. **e**, Pseudovirus-neutralization activities of XBB.1.5-neutralizing mAbs in groups A1 (n = 70, p < 0.0001) and A2 (n = 23, p < 0.0001) against XBB.1.5 and XBB.1.5.10; and mAbs in groups B (n = 15, p = 0.02) and C/D1 (n = 13, p = 0.001) against XBB.1.5 and XBB.1.16 (‘n’ refers to the number of mAbs). Fold changes in IC50 are labeled. P-values are calculated using two-tailed Wilcoxon signed-rank test of paired samples. **f**, Pseudovirus-neutralization activities of mAbs within the six crucial epitope groups (C/D1 [n = 76], D2 [n = 86], E1/E2.1 [n = 100], E2.2 [n = 124], E3 [n = 101], and F1 [n = 236], “n” refers to the number of mAbs) are shown against SARS-CoV-2 D614G, BA.5, BQ.1.1, and XBB.1.5. Geometric mean IC50 values are displayed as bars and labeled above each group of data points.

Using graph-based unsupervised clustering on the determined escape scores over sites on RBD, we identified 12 major epitope groups on the BA.5 RBD and embedded the monoclonal antibodies using uniform manifold approximation and projection (UMAP) for visualization (Fig. 4a). Names of the epitope groups were generally assigned in line with the epitope groups on WT RBD defined previously^3,32^. Neutralizing activities against SARS-CoV-2 D614G, BA.1, BA.2, BA.5, BA.2.75, BQ.1.1 and XBB.1.5 were determined using VSV-based pseudovirus-neutralization assays. In general, neutralization was highly correlated with targeting epitopes of monoclonal antibodies. Antibodies in epitope groups F3, A1, A2, B, C/D1, D2, D3, D4 and E1/E2.1 targeted neutralizing epitopes, whereas antibodies in the other three groups, E2.2, E3 and F1, exhibited weak or no neutralization activity (Fig. 4b and Extended Data Fig. 5b). Consistent with the plasma neutralization results, BA.5/BF.7 BTI plasma exhibited substantially imprinted antibody response, leading to more than 50% of antibodies targeting conserved weakly neutralizing epitopes. By contrast, convalescent individuals who experienced BA.5 or BF.7 reinfection after prior BA.1 or BA.2 BTI induced only around 20% of antibodies targeting such epitopes, indicating substantial alleviation of immune imprinting (Fig. 4c and Extended Data Fig. 5a). Of note, prior BA.1 or BA.2 BTI led to Omicron-specific antibodies targeting distinct epitopes after reinfection. Prior BA.1 BTI induced a higher level of group D3 antibodies, whereas BA.2 BTI cohorts had more antibodies in group F3, indicating that an Omicron infection history during repeated Omicron infections also introduced Omicron-based immune imprinting.

Among the 12 identified epitope groups, A1, D2, E1/E2.1, E2.2, E3 and F1 are similar to their corresponding WT-based groups and mainly consist of WT-reactive antibodies^32,41^ (Fig. 4d,e and Extended Data Fig. 5c,d). As expected, a BA.5-based epitope landscape also defines novel groups that mainly comprise Omicron-specific monoclonal antibodies, including groups A2, D3, D4 and F3. Notably, most antibodies in group F3 were not cross-reactive to WT RBD, which differs from the rare sarbecovirus-neutralizing broad NAbs in group F3 from individuals who had recovered from SARS, such as SA55 and BD55-3372^42^. Compared with group A1, which mainly contains IGHV3-53/3-66 public antibodies^43,44^ (also known as class 1 or site Ia), monoclonal antibodies in group A2 are susceptible to mutations on 417 and 505, including the reversions. Group D3 and D4 target an epitope near that of group D2 (targeted by LY-CoV1404), but exhibited distinct escape profiles or interacting residues^45^. D3 is susceptible to N439 and K440 mutations, and was thus escaped by WT owing to N440, whereas the footprint of D4 is closer to the receptor-binding motif (RBM), interacting with G447, Y449 and R498 (Fig. 4d,e). Antibodies in the WT-based groups B, C and D1 were mostly escaped by L452R, E484A and F486V in BA.5. Groups B and C/D1 here comprise both WT-reactive and Omicron-specific antibodies; group B is more focused on N487 and Y489, and C/D1 mainly focus on F490, which is largely escaped by F490S in XBB variants (Fig. 4d,e and Extended Data Fig. 5c,d). Among the 12 groups, A1, A2, B and D3, and especially D4 and F3, comprise a substantial proportion of NAbs exhibiting broad neutralization against BQ.1.1 and XBB.1.5 (Fig. 4f). Groups C/D1, D2 and E1/E2.1 also comprise a small proportion of XBB.1.5-neutralizing monoclonal antibodies (Extended Data Fig. 5f). Considering the recent emergence and prevalence of XBB subvariants with F456L (XBB.1.5.10) or K478R (XBB.1.16) substitutions, which are crucial sites for NAbs in groups A1 and A2 or B and C/D1, respectively, we tested the neutralization of XBB.1.5-neutralizing antibodies from these groups against these two mutants. As expected, F456L escapes or dampens the neutralization of most XBB.1.5-neutralizing antibodies in group A1 or A2, and XBB.1.16 (E180V/K478R) also escapes a large proportion of NAbs in groups B and C/D1 (Extended Data Fig. 5e). Overall, these results demonstrate that repeated Omicron infection stimulates a higher level of Omicron-specific NAbs targeting neutralizing epitopes compared with one-time Omicron BTI, indicating substantial alleviation of immune imprinting on the antibody epitope level. These Omicron-specific monoclonal antibodies have distinct RBD epitopes and escaping mutations compared to WT-induced monoclonal antibodies, introducing a large neutralizing epitope shift and contributing majorly to the broadly neutralizing capability against XBB lineages.

## Evolutionary hotspots on XBB.1.5 RBD

**Fig. 5:**
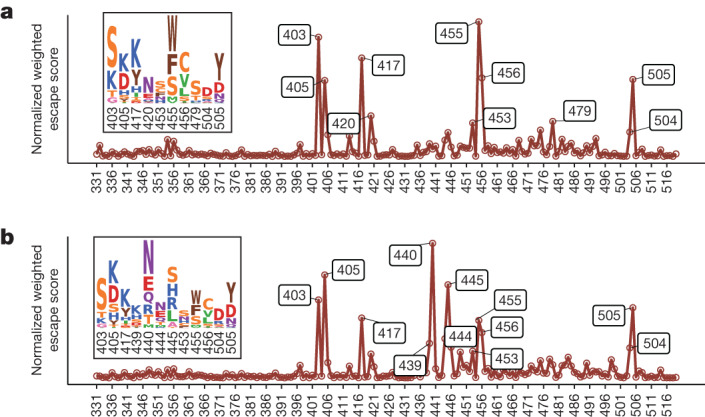
Estimation of the evolutionary trends of XBB.1.5 RBD from DMS profiles. **a**,**b**, Normalized average DMS escape scores weighted by IC_50_ against XBB.1.5 using DMS profiles of monoclonal antibodies from BA.5/BF.7 BTI (**a**), and monoclonal antibodies from BA.5/BF.7 BTI and BA.1/BA.2 BTI with BA.5/BF.7 reinfection (**b**). The effects of each mutation on ACE2 binding and RBD expression and the codon constraints on each residue are also considered (Methods). Residues with high estimated preferences are labelled, and their corresponding mutation scores are shown as logos.

**Extended Data Fig. 6:**
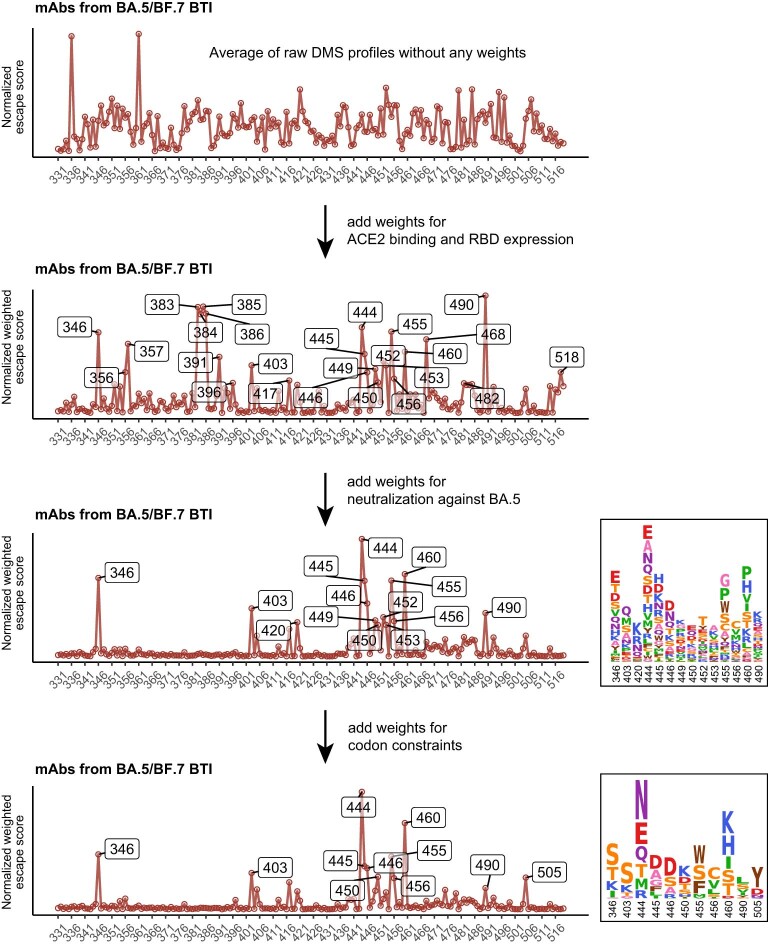
Workflow of calculating weighted escape scores of each mutation on RBD. Weights for ACE2 binding and RBD expression, neutralization activity, and codon usage are sequentially applied on the calculation to achieve informative results. Mutation preferences of BA.5 RBD under the pressure of NAbs from BA.5 or BF.7 BTI are shown.

Encouraged by the successful rationalization of the prevalence of F456L and K478R based on DMS, we aimed to systematically investigate the evolutionary preference for the XBB RBD. To integratively evaluate the preference of each mutation considering their effects on NAb escape, human ACE2 binding, RBD stability and codon constraints, we previously calculated a weighted preference score for RBD mutations using WT-based DMS profiles and neutralizing activities against BA.5 to predict the convergent evolution of the BA.5 RBD^3^ (Extended Data Fig. 6). We used a similar approach with BA.5-based DMS profiles and neutralization against XBB.1.5 to identify the evolutionary trends of the XBB.1.5 RBD. When considering antibodies from BA.5/BF.7 BTI only, the most important sites include R403S/K, N405K, N417Y, Y453S/C/F, L455W/F/S, F456C/V/L and H505Y/D, corresponding to escape hotspots of groups A1, A2 and F3 (Fig. 5a). With antibodies from repeated Omicron infection included in the analysis, scores of N439K, K440N/E, K444N/E and P445S/H/R/L become higher, corresponding to groups D3 and D4, which are consistent with the epitope distributions of monoclonal antibodies from each cohort (Fig. 5b). Notably, N405D and N417K reversions should hardly appear in the real world owing to the potential recovery of previously escaped NAbs in groups F2 and A, respectively. K478 mutations are not identified in the calculation, which is also a limitation of our model due to the low proportion of XBB-neutralizing antibodies in group B or C/D1 in our cohorts.

**Fig. 6:**
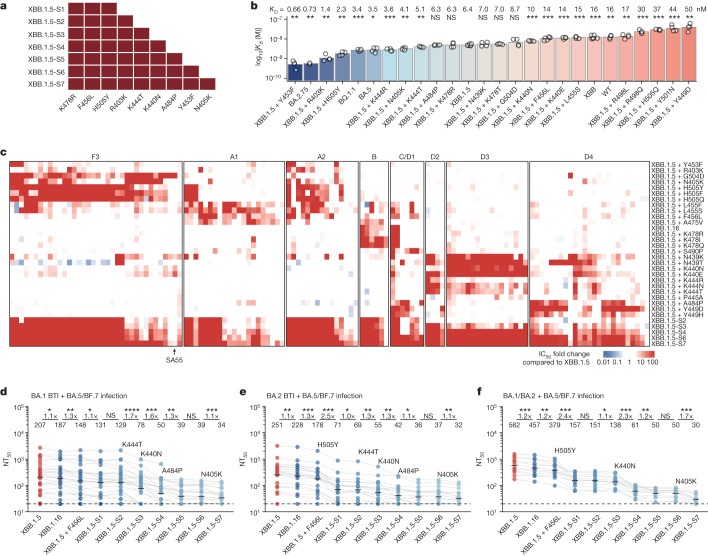
Combination of escape mutations evades XBB.1.5-neutralizing antibodies from reinfection. **a**, Generation of SARS-CoV-2 XBB.1.5-based pseudoviruses with combinations of critical mutations identified through analysis of DMS profiles. **b**, Human ACE2-binding affinity for various RBD mutants of SARS-CoV-2, assessed using SPR. Geometric mean dissociation constants (*K*_d_) from at least four independent replicates are shown. *P* values for the comparison with the *K*_d_ for XBB.1.5 RBD were determined using a two-tailed *t*-test on log-transformed *K*_d_ values and are shown above the bars. *n* = 2 for BA.2.75; *n* = 6 for XBB.1.5 and XBB.1.5 + F456L; and *n* = 4 for other groups. **c**, IC_50_ values for representative potent XBB.1.5-neutralizing antibodies from different epitope groups against XBB.1.5 variants carrying individual or multiple escape mutations. Fold changes in IC_50_ against the mutants relative to XBB.1.5 are presented as a heat map. **d**–**f**, NT_50_ for SARS-CoV-2 XBB.1.5-based mutants, using plasma from convalescent individuals who experienced BA.5 or BF.7 reinfection: BA.1 BTI prior to BA.5/BF.7 reinfection (*n* = 26) (**d**); BA.2 BTI prior to BA.5/BF.7 reinfection (*n* = 19) (**e**); or reinfection with BA.5 or BF.7 after BA.1 or BA.2 infection without vaccination (*n* = 12) (**f**). Key mutations diminishing neutralization are labelled above the corresponding lines. Dashed lines indicate the limit of detection (NT_50_ = 20). GMTs are shown above data points. Statistical tests were performed between neighbouring mutants; two-tailed Wilcoxon signed-rank tests on paired samples.

**Extended Data Fig. 7:**
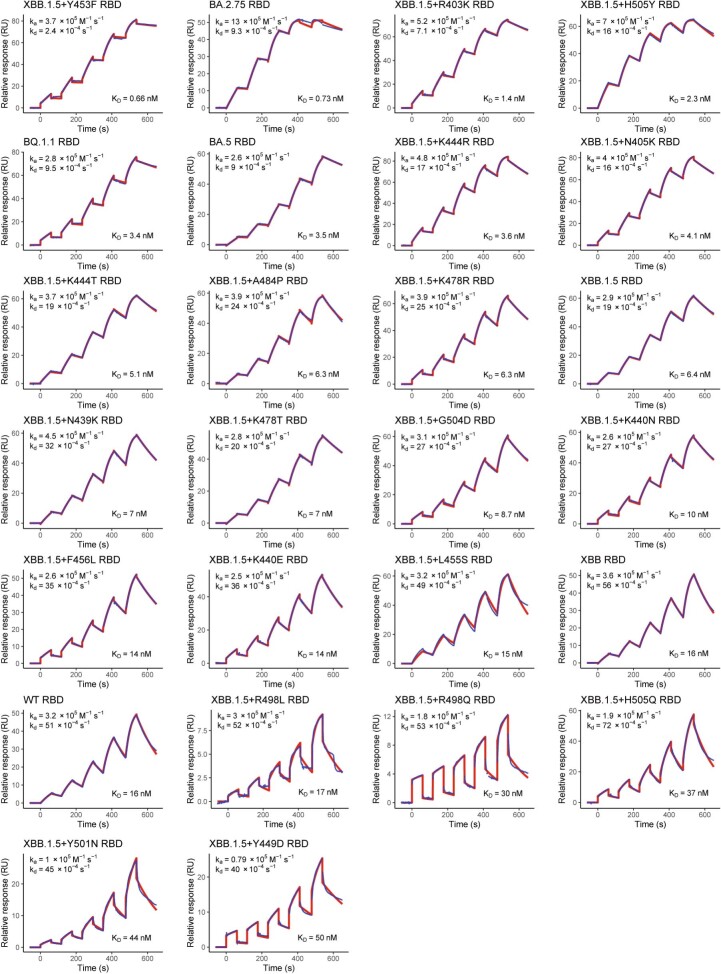
SPR sensorgrams for affinity of hACE2 and SARS-CoV-2 mutants RBD. Representative sensorgram of at least four replicates is shown for each RBD. Geometric mean kinetic constants k_a_, k_d_, and dissociation equilibrium constant K_D_ are labeled in each panel.

**Extended Data Fig. 8:**
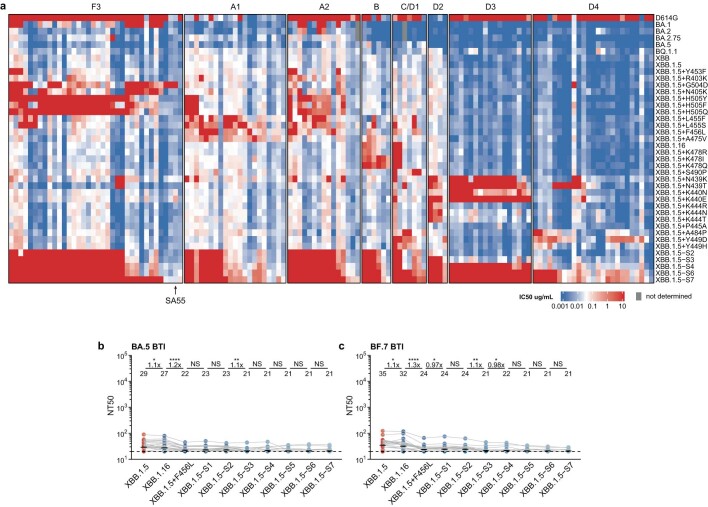
NAbs from BTI and reinfection are escaped by constructed mutants. **a**, IC50 values for representative potent XBB.1.5-neutralizing antibodies from different epitope groups against XBB.1.5 variants carrying individual or multiple escape mutations are shown. The order of antibodies is the same as that in Fig. 6c. **b**, Pseudovirus NT50 for SARS-CoV-2 XBB.1.5-based mutants are shown using plasma from convalescent individuals who experienced BA.5 (n = 36) or BF.7 BTI (n = 30). Statistical tests are performed between neighboring mutants. P-values are calculated using two-tailed Wilcoxon signed-rank tests on paired samples. *p < 0.05, **p < 0.01, ****p < 0.0001, and p > 0.05 (NS).

On the basis of the analysis above, we explored whether the combination of multiple escape mutations against major epitope groups effective against XBB.1.5 could essentially evade the broadly neutralizing capability of plasma from repeated Omicron infection while retaining high ACE2-binding affinity. Besides the two emerging mutations K478R and F456L, we selected seven additional substitutions, including H505Y, R403K, K444T, K440N, A484P, Y453F and N405K—which were sequentially added to XBB.1.5—and constructed seven pseudoviruses, XBB.1.5-S1 to XBB.1.5-S7 (Fig. 6a). The mutations were selected from a larger set of mutation candidates considering their impacts on human ACE2-binding affinity as determined by surface plasmon resonance (SPR) and the capability of escaping the neutralization of a panel of 131 potent XBB.1.5-neutralizing antibodies from 8 epitope groups (Fig. 6b,c and Extended Data Fig. 7a). XBB.1.5-S7 successfully escaped most of the NAbs in the panel, except for a small group of broad NAbs from group F3, A1 and D4, including SA55, a therapeutic antibody under clinical development^42^. We then evaluated the neutralization titres of convalescent plasma from individuals who experienced Omicron BTI or repeated Omicron infection against the designed escape mutants. As expected, XBB.1.5-S7 could significantly escape plasma samples from all tested cohorts. Plasma from BA.5 or BF.7 BTI were significantly escaped upon the inclusion of F456L, and were almost completely ineffective against XBB.1.5-S7 (Extended Data Fig. 8b). Plasma from repeated Omicron infections was much more resistant to escape mutations. Of note, plasma from BA.5/BF.7 reinfection with prior BA.1 BTI or BA.2 BTI exhibited distinct neutralization to different escape mutants. Plasma samples from BA.5/BF.7 reinfection with prior BA.1 BTI were largely evaded by K444T and K440N, but not strongly affected by H505Y, whereas those with prior BA.2 BTI were significantly evaded by H505Y (Fig. 6d,e). This is consistent with the observation that reinfection with prior BA.1 BTI elicits more group D3 antibodies, whereas reinfection with prior BA.2 BTI elicits more group F3 antibodies (Fig. 4c). Unvaccinated reinfection cohorts exhibited higher neutralization against XBB.1.5 compared with vaccinated cohorts, but were equivalently escaped by XBB.1.5-S7. The most significant reduction in neutralization occurred upon the inclusion of H505Y, K440N and N405K, indicating a high proportion of Omicron-specific antibodies in groups D3 and F3 (Fig. 6f).

## Discussion

In summary, our findings suggest that secondary Omicron exposure is necessary to mitigate the immune imprinting conferred by previous ancestral virus exposure and to elicit higher levels of Omicron-specific antibodies. Accordingly, our recommendation is to administer two booster doses of Omicron-based vaccines to individuals who have not received prior Omicron-based vaccinations or have not been previously infected with the Omicron variant. Moreover, administering the second booster shot after a prolonged interval can provoke a wider and more efficient immune response, whereas incorporating WT virus into subsequent vaccine designs may worsen outcomes^26^.

Recently, several fast-growing XBB lineages, such as the variant of interest XBB.1.16, have acquired RBD mutations on K478. However, the K478 mutation did not emerge in our prediction of evolutionary trends for XBB.1.5 RBD. This contradiction may be attributed to the fact that our mutational prediction model relies primarily on the cohorts that we recruited, and we did not capture the immune background that introduced K478 mutation. One possible background that may give rise to K478 is repeated BA.5/BQ.1.1/XBB exposure, as F486 could mask the immunogenicity of K478. Another potential source of K478 is Delta-imprinted convalescent individuals who experienced BA.5/BQ.1.1/XBB infections, which could result in the generation of abundant K478X-sensitive monoclonal antibodies, given that Delta carries T478K. This may explain why K478X is observed mostly in India^8,46^.

The degree of immune imprinting might be different between mRNA and inactivated virus vaccinations. Recent studies have shown that subsequent exposure to Omicron twice after two doses of WT-based mRNA vaccines still produce significantly low levels of Omicron-specific antibodies, despite the enhanced neutralization breadth against BQ.1.1 and XBB variants^47,48^. Additionally, individuals who have received two doses of mRNA vaccines and experienced two rounds of Omicron infection also have low levels of Omicron-specific antibodies^47^. This indicates that mRNA vaccines may generate a stronger immune imprinting effect compared with inactivated vaccines, potentially owing to its stronger humoral immune response^4,49^. However, a direct comparison is needed for validation.

## Methods

### Isolation of PBMCs and plasma

Blood samples from vaccinated or unvaccinated individuals who had recovered from Omicron breakthrough infection or reinfection were obtained under study protocols approved by Beijing Ditan Hospital, Capital Medical University (Ethics committee archiving no. LL-2021-024-02) and the Tianjin Municipal Health Commission, and the Ethics Committee of Tianjin First Central Hospital (Ethics committee archiving no. 2022N045KY). All participants have provided written informed consent for the collection of information, storage and use of their clinical samples for research purposes and publication of data generated from this study.

Samples from one-time breakthrough infection and the first infections in repeat-infection cohorts were collected during the ‘zero COVID’ period in China. During that period, the total number of infected individuals was small and there were clear epidemiological correlations between confirmed cases. BA.1 breakthrough infections occurred in Tianjin in January and a cumulative count of 430 individuals tested positive for Omicron BA.1 by 7 February 2022, with no additional infections identified in the subsequent 16 days^38^. BA.2 breakthrough infections occurred in Beijing between April and July 2022. From 22 April to 14 November, a total of 2,230 cases of local infections were reported in Beijing, and BA.2.2.1 (BA.2 + I1221T in spike) was the most prevalent subvariant in Beijing between April and July^50^. BA.5 breakthrough infections occurred in Beijing and Tianjin between September and October 2022^50^. BF.7 breakthrough infections occurred in Inner Mongolia in November 2022, and BF.7 accounted for 100% of the sequences^51^. These samples of infection were confirmed by PCR, and the majority also underwent sequencing to determine the viral strains. The unsequenced samples, which make up only a small proportion of the total samples, showed strong epidemiological correlations with the sequenced samples.

Reinfections were confirmed by PCR or antigen testing. While the viral strain types for these infections were not confirmed through sequencing, it is important to note that these samples were confirmed in December 2022 in Beijing and Tianjin. At that time, these regions were predominantly undergoing the BA.5/BF.7 wave^50^. Among the sequences from samples collected between 1 December 2022 and 2 January 2023, >98% of them were designated as BA.5* (excluding BQ*). Specifically, the major subtypes circulating in China at that time were BA.5.2.48* (DY*) and BF.7.14*, which do not harbour additional mutations on RBD, and thus can be generally considered as BA.5/BF.7 in this study (cov-spectrum.org/explore/China/AllSamples/from%3D2022-12-01%26to%3D2023-02-01/variants?&).

The whole blood samples were 1:1 diluted with 2% fetal bovine serum (FBS) (Hyclone, SH30406.05) in phosphate buffered saline (PBS) (Invitrogen, C10010500BT) and subjected to Ficoll (Cytiva, 17-1440-03) gradient centrifugation to isolate plasma and PBMCs. Plasma was collected from upper layer after centrifugation. PBMCs were collected at the interface and further prepared through centrifugation, red blood cell lysis (Invitrogen eBioscience 1× RBC Lysis Buffer, 00-4333-57) and washing steps. If not used for downstream process immediately, samples were stored in FBS with 10% DMSO (Sigma-Aldrich, D4540) in liquid nitrogen. All PBMC samples were shipped on dry ice and cryopreserved PBMCs were thawed in PBS  +  1 mM EDTA (Invitrogen, AM9260G) + 2% FBS before use.

### mRNA and protein vaccine preparation and mouse immunization

For mRNA vaccine preparation, 5′ untranslated region (UTR), target sequence, and 3′ UTR were sequentially inserted after T7 promoter in an empty PSP73 plasmid first. The plasmid was then subjected to double digestion to obtain linearized DNA. This DNA served as a template for an in vitro transcription reaction mediated by T7 RNA polymerase to synthesize RNA encoding the SARS-CoV-2 S6P (F817P, A892P, A899P, A942P, K986P, V987P, R683A and R685A) protein according to the manufacturer’s instructions (Vazyme, DD4201). Transcription products were treated with DNase I to remove DNA templates, and purified using VAHTS RNA Clean Beads (Vazyme, N412-02). Cap 1 structure was added using Vaccinia Capping Enzyme (Vazyme, DD4109) and mRNA Cap 2′-*O*-methyltransferase (Vazyme, DD4110), followed by magnetic bead purification. Poly(A) tails were added using *Escherichia coli* Poly(A) Polymerase (Vazyme, N4111-02) and the product was purified again.

The mRNA was encapsulated in a functionalized lipid nanoparticle as described previously^52^. In brief, ionizable lipid, DSPC, cholesterol, and PEG2000-DMG were dissolved in ethanol at the mole ratio of 50:10:38.5:1.5, respectively. mRNA was diluted in RNase free 50 mM citrate buffer (pH 4.0) to obtain a final lipid:mRNA weight ratio of 6:1. The aqueous and ethanol solutions were mixed in a 3:1 volume ratio using a microfluidic apparatus and the obtained lipid nanoparticles were dialysed overnight. All of the samples were stored within a week at 2 ~ 8 °C of use to ensure the chemical stability of the components. The size of lipid nanoparticles, the particle size distributions, and the encapsulation and concentration of mRNA were determined. The encapsulation in all of the samples was typically 90–99%.

The spike proteins, including D614G (ACROBiosystems, SPN-C52H9), XBB (ACROBiosystems, SPN-C5248), BQ.1.1 (ACROBiosystems, SPN-C522s), BA.1 (ACROBiosystems, SPN-C522a) and BA.5 (ACROBiosystems, SPN-C522e) were used for mouse immunization. All of these proteins were modified to incorporate 6P2A mutations (F817P, A892P, A899P, A942P, K986P, V987P, R683A and R685A) and a T4 fibritin foldon domain at the C terminus to improve the stability of the trimeric structure.

Animal experiments were carried out under study protocols approved by Rodent Experimental Animal Management Committee of Institute of Biophysics, Chinese Academy of Sciences (SYXK2023300) and Animal Welfare Ethics Committee of HFK Biologics (HFK-AP-20210930). Six- to eight-week-old female BALB/c mice were used for experiments. The mice were kept under a 12-hour light and 12-hour dark cycle, with room temperatures maintained between 20 °C and 26 °C. The humidity levels in the housing area ranged from 30% to 70%. Mice were immunized according to schemes in Fig. 1. All inactivated vaccines were administered intraperitoneally at a dose of 3 μg per mouse, while mRNA vaccines were administered intramuscularly at a dose of 1 μg or 10 μg per mouse. Protein subunit vaccines were administered subcutaneously at six sites on the back at a dose of 10 μg per mouse, where complete Freund’s adjuvant was used for the prime immunization, and incomplete Freund’s adjuvant was used for booster immunizations, with a 1:1 volume ratio of protein subunit and adjuvant. The second immunizations were given 2 weeks after the first dose, with subsequent doses administered at 1-month intervals, unless stated otherwise. Blood samples were collected 4 week after the final immunization.

### BCR sequencing, analysis and recombinant antibody expression

CD19^+^ B cells were enriched from PBMCs using EasySep Human CD19 Positive Selection Kit II (STEMCELL, 17854). Following enrichment, every 1 × 10^6^ B cells in 100 μl buffer were incubated with a panel of antibodies including 3 μl FITC anti-human CD20 antibody (BioLegend, 302304), 3.5 μl Brilliant Violet 421 anti-human CD27 antibody (BioLegend, 302824), 2 μl PE/Cyanine7 anti-human IgD antibody (BioLegend, 348210) and 2 μl PE/Cyanine7 anti-human IgM antibody (BioLegend, 314532). Additionally, fluorophore or oligonucleotide conjugated RBD were added. For FACS, 0.013 μg of biotinylated BA.1 (Sino Biological, 40592-V49H7-B) or BA.2 (customized from Sino Biological) RBD protein conjugated with PE-streptavidin (BioLegend, 405204) and APC-streptavidin (BioLegend, 405207), and 0.013 μg of WT biotinylated RBD protein (Sino Biological, 40592-V27H-B) conjugated with BV605-streptavidin (BioLegend, 405229) were added. For sequencing, BA.1 or BA.2 biotinylated RBD protein conjugated with TotalSeq-C0971 Streptavidin (BioLegend, 405271) and TotalSeq-C0972 Streptavidin (BioLegend, 405273), WT biotinylated RBD protein conjugated with TotalSeq-C0973 Streptavidin (BioLegend, 405275) and TotalSeq-C0974 Streptavidin (BioLegend, 405277) and biotinylated Ovalbumin (Sino Biological) conjugated with TotalSeq-C0975 Streptavidin (BioLegend, 405279) were added. After incubation and washing steps, 5 μl of 7-AAD (Invitrogen, 00-6993-50) was included for dead cell exclusion.

Cells negative for 7-AAD, IgM and IgD, but positive for CD20, CD27 and BA.1 or BA.2 RBD were sorted using a MoFlo Astrios EQ Cell Sorter (Beckman Coulter). FACS data were collected by Summit 6.0 (Beckman Coulter) and analysed using FlowJo v10.8 (BD Biosciences).

The sorted B cells were processed using the Chromium Next GEM Single Cell V(D)J Reagent Kits v1.1 according to the manufacturer’s user guide (10X Genomics, CG000208). In brief, the cells were resuspended in PBS + 10% FBS after centrifugation and then processed to obtain gel beads-in-emulsion (GEMs) using the 10X Chromium controller. The GEMs were subjected to reverse transcription and the products were further purified with a GEM-RT clean up procedure. Preamplification was then performed on the products which were subsequently purified using the SPRIselect Reagent Kit (Beckman Coulter, B23318). The paired V(D)J BCR sequences were enriched with 10X BCR primers, followed by library preparation. Finally, the libraries were sequenced using the Novaseq 6000 platform, running either the Novaseq 6000 S4 Reagent Kit v1.5300 cycles (Illumina, 20028312).

The 10X Genomics V(D)J sequencing data were assembled as BCR contigs and aligned using the Cell Ranger (v6.1.1) pipeline according to the GRCh38 BCR reference. To ensure high quality, only the productive BCR contigs and cells with one heavy chain and one light chain were retained. The IgBlast program (v1.17.1) and Change-O toolkit (v1.2.0) were utilized to annotate the germline V(D)J genes and detect somatic hypermutation sites in the variable domain of the BCR sequences.

For expression optimization in human cells, heavy and light chain genes were synthesized by GenScript, inserted separately into plasmids (pCMV3-CH, pCMV3-CL or pCMV3-CK) via infusion (Vazyme, C112), and co-transfected into Expi293F cells (Thermo Fisher, A14527) using polyethylenimine transfection. The cells were cultured at 36.5 °C in 5% CO_2_ and 175 rpm for 6–10 days. The cell expression fluid was collected and centrifuged. After centrifugation, supernatants containing the monoclonal antibodies were purified using protein A magnetic beads (Genscript, L00695). The purified samples were determined by SDS–PAGE.

### Pseudovirus-neutralization assay

Codon-optimized SARS-CoV-2 *S* gene was inserted into the pcDNA3.1 vector to construct plasmids encoding the spike proteins of SARS-CoV-2. The 293 T cell line (ATCC, CRL-3216) was transfected with the spike protein-expressing plasmids and then infected with G*ΔG-VSV virus (Kerafast, EH1020-PM). After culturing, the pseudovirus-containing supernatant was collected, filtered, aliquoted, and frozen at −80 °C for future use. Pseudovirus-neutralization assays were conducted on the Huh-7 cell line (Japanese Collection of Research Bioresources (JCRB), 0403).

Monoclonal antibodies or plasma were serially diluted in DMEM (Hyclone, SH30243.01) and incubated with pseudovirus in 96–well plates at 5% CO_2_ and 37 °C for 1 h. Digested Huh-7 cell (JCRB, 0403) were seeded and cultured for 24 h. Half of the supernatant was then discarded and Bright-Lite Luciferase Assay Substrate (lyophilized) was mixed with Bright-Lite Luciferase Assay Buffer (Vazyme, DD1209-03-AB) and then the mixture was added to react in the dark. The luminescence value was detected using a microplate spectrophotometer (PerkinElmer, HH3400). IC_50_ was determined by a four-parameter logistic regression model using PRISM (version 9.0.1).

### Authentic virus neutralizing assay

The serum samples obtained from Convalescent individuals were heat-inactivated at 56 °C for 0.5 h and subsequently diluted in twofold steps with cell culture medium. These diluted sera were mixed with a virus suspension (SARS-CoV-2 Wuhan, BA.1(GISAID, EPI_ISL_8187354), BA.5.2.1 (GISAID, EPI_ISL_17261619.2), BF.7.14 (GISAID, EPI_ISL_17959240), FL.8 (XBB.1.9.1.8) (GISAID, EPI_ISL_17262369) containing 100 cell culture infectious dose 50% (CCID_50_) and added to 96-well plates at a 1:1 ratio. The plates were then incubated at 36.5 °C in a 5% CO_2_ incubator for 2 h. Following the incubation period, Vero cells (Gifted from WHO, (ATCC, CCL-81)) were added to each well containing the serum–virus mixture. The plates were further incubated for 5 days at 36.5 °C in a 5% CO_2_ incubator. Microscopic observation of cytopathic effects was performed, and the neutralizing titre was determined based on the highest dilution that showed 50% protection against the virus-induced cytopathic effects. Experiments were conducted in a biosafety level 3 (ABSL3) facility.

### ELISA

ELISA assays were conducted by pre-coating ELISA plates with RBD (SARS-CoV-2 WT, SARS-CoV-2 BA.1, SARS-CoV-2 BA.2 RBD, Sino Biological) at concentrations of 0.03 μg ml^−1^ and 1 μg ml^−1^ in 0.05 M coating buffer (Solarbio, C1055) overnight at 4 °C. The plates were then washed and blocked, after which 100 μl of 1 μg ml^−1^ antibodies were added to each well and incubated at room temperature for 30 mins. Following incubation, the plates were washed and incubated with 0.25 μg ml^−1^ Peroxidase-conjugated AffiniPure goat anti-human IgG (H + L) (JACKSON, 109-035-003) for 30 min at room temperature. The reaction was developed using tetramethylbenzidine (TMB) (Solarbio, PR1200), and stopped by adding H_2_SO_4_. The absorbance was measured at 450 nm using a microplate reader (Thermo Scientific, Multiskan Fc) and the negative control used was the 1 μg ml^−1^ H7N9 human IgG1 antibody HG1K (Sino Biological, HG1K).

### Surface plasmon resonance

Human ACE2 with Fc tag was immobilized onto protein A sensor chips using a Biacore 8 K (GE Healthcare). Purified SARS-CoV-2 mutants RBD were prepared in serial dilutions, ranging from 100 to 6.25 nM, and injected over the sensor chips. The response units were recorded at room temperature using BIAcore 8 K Evaluation Software (v4.0.8.20368; GE Healthcare). The obtained data were then analysed using BIAcore 8 K Evaluation Software (v4.0.8.20368; GE Healthcare) and fitted to a 1:1 binding model.

### DMS library construction

Duplicate single site saturated mutant libraries spanning all 201 amino acids of BA.5 RBD (position N331-T531 by Wuhan-Hu-1 reference numbering) were constructed based on previously reported method, in order to ensure the reproducibility and reliability of results^53^. A unique N26 barcode was PCR appended to each RBD variant as an identifier, and the correspondence of variant and N26 barcode was obtained by PacBio sequencing on Sequel ll platform. The BA.5 RBD mutant libraries were assembled into pETcon 2649 vector and amplified in DH10B cells. Above plasmids products were then transformed into *Saccharomyces cerevisiae* EBY100. Yeasts were screened on SD-CAA plates and further enlarged in SD-CAA liquid media, the resulted libraries were preserved at −80 °C after flash frozen in liquid nitrogen.

### MACS-based mutation escape profiling

The high-throughput mutation escape profiling for every single antibody was performed as previously described^3,32^. In brief, non-functional RBD variants were first eliminated from BA.5 mutant libraries by magnetic-activated cell sorting (MACS). The selected yeasts were inoculated into SG-CAA media to induce RBD surface expression by overnight culture. To capture yeast cells that escape specific antibody binding, two rounds of sequential negative selection and one round of positive selection were carried out based on MACS. After overnight amplification, plasmids were extracted from the sorted yeasts using the 96-Well Plate Yeast Plasmid Preps Kit (Coolaber, PE053), then used as template for N26 barcode amplification by PCR. Final PCR products were purified, quantified, and sequenced on Nextseq 500 or Nextseq 550 platform.

### DMS data analysis and antibody clustering

DMS raw sequencing data were processed as described previously^3,32^. In brief, the detected barcode sequences of both the antibody-screened and reference library were aligned to the barcode-variant dictionary generated using dms_variants (v0.8.9) from PacBio sequencing data of the BA.5 DMS library. Only barcodes that are detected more than 5 times in the reference library are included in the calculation to avoid large sampling error. The escape scores of a variant X that are detected both in the screened and reference library were defined as *F* × (*n*_*X*,ab_/*N*_ab_)/(*n*_*X*,ref_/*N*_ref_), where *F* is a scale factor to normalize the scores to the 0–1 range, while *n* and *N* are the number of detected barcodes for variant *X* and total barcodes in antibody-screened (ab) or reference (ref) samples, respectively. To assign an escape score to each single substitution on RBD, an epistasis model is fitted using dms_variants (v0.8.9) as described previously^53,54^. For antibodies with multiple replicates of DMS, the final escape score of each mutation is the average over all replicates.

We used graph-based unsupervised clustering and embedding to assign an epitope group for each antibody and visualize them in a two-dimensional space. First, site escape scores (the sum of mutation escape scores on a residue) of each antibody are first normalized to a sum of one and considered as a distribution over RBD residues. The dissimilarity of two antibodies is defined by the Jessen-Shannon divergence of the normalized escape scores. Pair-wise dissimilarities of all antibodies in the dataset are calculated using the SciPy module (scipy.spatial.distance.jensenshannon, v1.7.0). Then, a 12-nearest-neighbour graph is built using python-igraph module (v0.9.6). Leiden clustering is performed to assign a cluster to each antibody^55^. The name of each cluster is annotated manually based on the featured sites on the average escape profiles of a cluster to make it consistent with the definition of our previously published DMS dataset using WT-based library in general^3^. To project the dataset onto a 2D space for visualization, we performed UMAP based on the constructed *k*-nearest-neighbour graph using umap-learn module (v0.5.2). Figures were generated by R package ggplot2 (v3.3.3).

### Estimate the preference of RBD mutations

Similar to the approach in our previous study^3^, we incorporated four types of weights in our calculations to account for the impact of each mutation on human ACE2-binding affinity, RBD expression, neutralizing activity, and the codon constraints on each residue. The weights for ACE2 binding and RBD expression are determined by tanh(*S*_bind_) + 1 and tanh(min(0, *S*_expr_)) + 1, respectively, where the *S*_bind_ and *S*_expr_ values are from the BA.2-based DMS on ACE2 binding and RBD expression^56^. The function tanh(*x*) is used as a sigmoidal curve to constrain the weights between 0 and 2. For codon constraint weights, mutations that cannot be accessed through single nucleotide mutation are first assigned a weight of zero. To address the intrinsic disparities in the frequency of distinct nucleotide substitutions in SARS-CoV-2, we assign different weights for mutations corresponding to various nucleotide substitutions^57^. Specifically, the weight of the most frequent substitution (C>T) is assigned a value of 0.1, while weights for G>T and G>A are 0.041 and 0.035, respectively. To retain the potential of rare mutations, all other substitutions are assigned a weight of 0.03. We use BA.4/5 (EPI_ISL_11207535) and XBB.1.5 (EPI_ISL_17054053) to define weights for codon usage. Regarding the neutralizing activities, the weight is calculated as −log_10_(IC_50_). IC_50_ values (μg ml^−1^) less than 0.0005 or greater than 1.0 are considered as 0.0005 or 1.0, respectively. As the dataset specifically enriches for Omicron-specific antibodies, potentially introducing bias when estimating mutation preferences. An additional weighting strategy is applied that assigns higher weights to cross-reactive monoclonal antibodies, resulting in 89% cross-reactive monoclonal antibodies for BA.5/BF.7 BTI cohorts and 51% for reinfection cohorts, as determined by unbiased characterization of monoclonal antibodies using ELISA. The raw escape scores for each antibody are first normalized by the maximum score among all mutants. The weighted score for each antibody and each mutation is obtained by multiplying the normalized scores with the corresponding four weights, and the final mutation-specific weighted score is the sum of scores for all antibodies in the designated set, which is then normalized once more to produce a value between 0 and 1. To visualize the calculated escape maps, sequence logos were created using the Python module logomaker (v0.8).

### Reporting summary

Further information on research design is available in the Nature Portfolio Reporting Summary linked to this article.

## Online content

Any methods, additional references, Nature Portfolio reporting summaries, source data, extended data, supplementary information, acknowledgements, peer review information; details of author contributions and competing interests; and statements of data and code availability are available at 10.1038/s41586-023-06753-7.

### Supplementary information

Supplementary Data 1 Flow cytometry gating scheme for antigen-specific memory B cell analysis and sorting.

Reporting Summary

Supplementary Table 1 Summarized information of SARS-CoV-2 convalescents involved in the study.

Supplementary Table 2 The epitopes, neutralizing activities, V(D)J germline genes and somatic hypermutations of 1,816 SARS-CoV-2 RBD-targeting antibodies from various immune backgrounds.

### Source data

Source Data Fig. 1

Source Data Extended Data Fig. 1

Source Data Extended Data Fig. 2

## Data Availability

Information on SARS-CoV-2 RBD-targeting monoclonal antibodies is included in Supplementary Table 2. Raw sequencing data for DMS are available on Genome Sequence Archive (GSA) of China National Center for Bioinformation with Project accession PRJCA020116. The Protein Data Bank model 7XNS is used for the structural model of SARS-CoV-2 RBD. Source data are provided with this paper.

## Code availability

Processed mutation escape scores and custom scripts for processing and analysing DMS data can be downloaded at github.com/jianfcpku/SARS-CoV-2-reinfection-DMS. The scripts have also been uploaded to Zenodo at 10.5281/zenodo.8373447.
