# Supplementary material for: Repeated Omicron exposures override ancestral SARS-CoV-2 immune imprinting
Source: Nature. 2023 Nov 22;625(7993):148–56. doi: 10.1038/s41586-023-06753-7 (PMC10764275; doi:10.1038/s41586-023-06753-7)
Supplement: Supplementary file 1 — Flow cytometry gating scheme for antigen-specific memory B cell analysis and sorting. [file 41586_2023_6753_MOESM1_ESM.pdf]

nature portfolio

https://doi.org/10.1038/s41586-023-06753-7

Supplementary information

Repeated Omicron exposures override ancestral SARS-CoV-2 immune imprinting

In the format provided by the authors and unedited

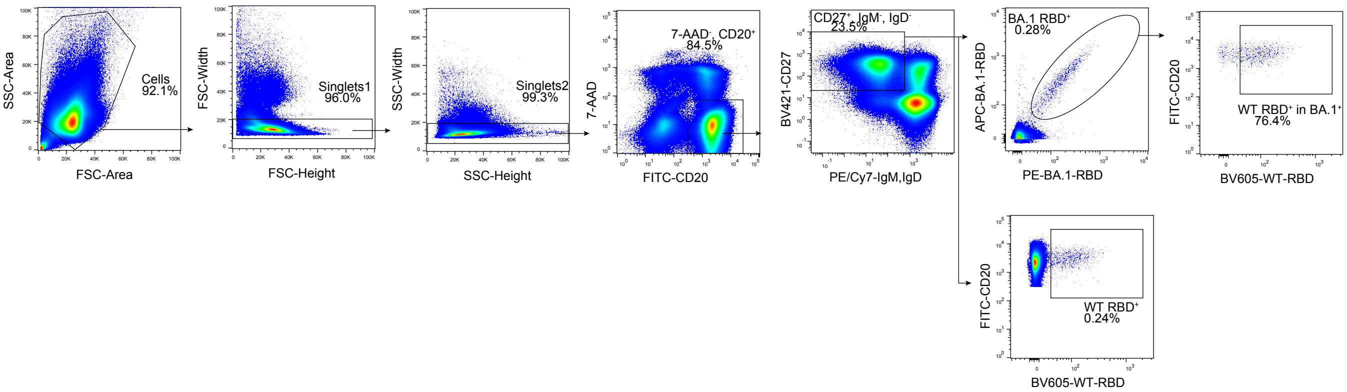

Supplementary Data 1

Flow cytometry gating strategy for antigen-specific memory B cell analysis and sorting. Gating was on singlets that were 7-AAD-, CD20+, IgM-, IgD-, CD27+, and Omicron (BA.1 or BA.2) RBD+. Within the Omicron RBD+ population, cells positive for WT RBD were further gated. Sorted cells were BA.1 RBD+ for BA.1 convalescents and BA.2 RBD+ for BA.2 convalescents. As an example, the gating of BA.1 RBD+ cells from BA.1 BTI individuals in Figure 3a is shown here. Other plots in Figure 3a-d were gated with the same strategy.
