# Supplementary material for: Repeated Omicron exposures override ancestral SARS-CoV-2 immune imprinting
Source: Nature. 2023 Nov 22;625(7993):148–56. doi: 10.1038/s41586-023-06753-7 (PMC10764275; doi:10.1038/s41586-023-06753-7)
Supplement: Supplementary file 2 — Reporting Summary [file 41586_2023_6753_MOESM2_ESM.pdf]

nature portfolio

Corresponding author(s): Yunlong Cao

Last updated by author(s): 11/13/2023

Reporting Summary

Nature Portfolio wishes to improve the reproducibility of the work that we publish. This form provides structure for consistency and transparency in reporting. For further information on Nature Portfolio policies, see our Editorial Policies and the Editorial Policy Checklist.

Statistics

For all statistical analyses, confirm that the following items are present in the figure legend, table legend, main text, or Methods section.

n/a	Confirmed	
☐	☒	The exact sample size (*n*) for each experimental group/condition, given as a discrete number and unit of measurement
☐	☒	A statement on whether measurements were taken from distinct samples or whether the same sample was measured repeatedly
☐	☒	The statistical test(s) used AND whether they are one- or two-sided<br>*Only common tests should be described solely by name; describe more complex techniques in the Methods section.*
☒	☐	A description of all covariates tested
☒	☐	A description of any assumptions or corrections, such as tests of normality and adjustment for multiple comparisons
☐	☒	A full description of the statistical parameters including central tendency (e.g. means) or other basic estimates (e.g. regression coefficient) AND variation (e.g. standard deviation) or associated estimates of uncertainty (e.g. confidence intervals)
☐	☒	For null hypothesis testing, the test statistic (e.g. *F*, *t*, *r*) with confidence intervals, effect sizes, degrees of freedom and *P* value noted<br>*Give P values as exact values whenever suitable.*
☒	☐	For Bayesian analysis, information on the choice of priors and Markov chain Monte Carlo settings
☒	☐	For hierarchical and complex designs, identification of the appropriate level for tests and full reporting of outcomes
☒	☐	Estimates of effect sizes (e.g. Cohen's *d*, Pearson's *r*), indicating how they were calculated

Our web collection on statistics for biologists contains articles on many of the points above.

Software and code

Policy information about availability of computer code

Data collection	Pseudovirus neutralization and ELISA data were collected by Multiskan™ FC Microplate Photometer.<br>SPR data was collected by BIAcore 8K Evaluation Software (v4.0.8.20368; GE Healthcare).<br>FACS data was collected by Summit 6.0 (Beckman Coulter).
Data analysis	Neutralization assays data were analyzed using PRISM (v9.0.1) .<br>FACS data were analyzed by FlowJo 10.8.<br>SPR data were analyzed by BIAcore 8K Evaluation Software ((v4.0.8.20368; Cytiva).<br>Sequence alignment of Omicron sublineages was performed by biopython (v1.78); V(D)J sequence data were aligned using Cell Ranger (v6.1.1), The IgBlast program (v1.17.1) and Change-O toolkit (v1.2.0) were utilized to annotate the germline V(D)J genes and detect somatic hypermutation sites in the variable domain of the BCR sequences.<br>Illumina barcodes sequencing data from deep mutational scanning experiments were analyzed using custom scripts (https://github.com/jianfcpku/SARS-CoV-2-reinfection-DMS) and Python package dms_variants (v0.8.9).<br>Custom scripts to analyze the escape mutation profiles data are available at Zenodo (DOI: 10.5281/zenodo.8373447) and Github (https://github.com/jianfcpku/SARS-CoV-2-reinfection-DMS).<br>We used Python package logomaker (v0.8), R package ggseqlogo (v0.1) and ggplot2 (v3.3.3) for illustration.<br>We utilized Python package python-igraph (v0.9.6), scipy (v1.7.0), scikit-learn (v0.24.2), leidenalg (v0.8.7), umap-learn (v0.5.2) to perform clustering and UMAP embedding fo antibodies. 2D UMAP plots are generated by ggplot2 (v3.3.3).

For manuscripts utilizing custom algorithms or software that are central to the research but not yet described in published literature, software must be made available to editors and reviewers. We strongly encourage code deposition in a community repository (e.g. GitHub). See the Nature Portfolio guidelines for submitting code & software for further information.

Data

Policy information about availability of data

All manuscripts must include a data availability statement. This statement should provide the following information, where applicable:

- Accession codes, unique identifiers, or web links for publicly available datasets
- A description of any restrictions on data availability
- For clinical datasets or third party data, please ensure that the statement adheres to our policy

Sequences and neutralization of the antibodies are included in Supplementary Table 2. Raw sequencing data of DMS assays are available on Genome Sequence Archive (GSA) of China National Center for Bioinformation (https://ngdc.cncb.ac.cn/gsa) with Project accession PRJCA020116. We used vdj_GRCh38_alts_ensembl-5.0.0 as the reference of V(D)J alignment, which can be obtained from https://support.10xgenomics.com/single-cell-vdj/software/downloads/latest. PDB 7XNS is used for the structural model of SARS-CoV-2 BA.5 RBD.

Field-specific reporting

Please select the one below that is the best fit for your research. If you are not sure, read the appropriate sections before making your selection.

☒ Life sciences ☐ Behavioural & social sciences ☐ Ecological, evolutionary & environmental sciences

For a reference copy of the document with all sections, see nature.com/documents/nr-reporting-summary-flat.pdf

Life sciences study design

All studies must disclose on these points even when the disclosure is negative.

Sample size

A total of 1816 antibodies were characterized in the manuscript. We analyzed all antibodies in hand and the sample size of antibodies in this study was sufficient to reach statistical significance by two-tailed binomial test for the differences in epitope distribution.
We collected plasma samples from 50 convalescent individuals with BA.1 breakthrough infection, 22 long-term convalescent individuals with BA.1 breakthrough infection, 39 with BA.2 breakthrough infection, 36 with BA.5 breakthrough infection and 30 with BF.7 breakthrough infection, all of whom received three doses of CoronaVac before infection. Further, we investigated 26 individuals who had post-vaccination BA.1 breakthrough infection followed by BA.5/BF.7 reinfection, 19 with post-vaccination BA.2 breakthrough infection followed by BA.5/BF.7 reinfection, and 12 individuals with BA.1/BA.2 infection followed by BA.5/BF.7 reinfection, who had no history of vaccination.
We analyzed all plasma samples collected and the sample size of plasma could reach statistical significance of NT50 values from neutralization assays by two-tailed Wilcoxon signed-rank test.
No sample size calculation was performed.

Data exclusions

466 antibodies were excluded from the study because of insufficient antibody or failed deep mutational scanning experiments, which is defined as no mutations scored two times of the median score.

Replication

Experimental assays were performed in at least two independent experiments according to or exceeding standards in the field. Specifically, we performed mutation screening using two independently constructed mutant libraries. We conducted all neutralization assays and ELISA in at least two independent experiments. All replicates for neutralization and ELISA are successful.

Randomization

Randomization was not required since we were applying a uniform set of measurements across the panel of monoclonal antibodies and plasma. As this is an observational study, randomization is not relevant.

Blinding

Blinding was not required since we were applying a uniform set of measurements across the panel of monoclonal antibodies and plasma. As this is an observational study, investigators were not blinded.

Reporting for specific materials, systems and methods

We require information from authors about some types of materials, experimental systems and methods used in many studies. Here, indicate whether each material, system or method listed is relevant to your study. If you are not sure if a list item applies to your research, read the appropriate section before selecting a response.

Materials & experimental systems

n/a	Involved in the study	
☐	☒	Antibodies
☐	☒	Eukaryotic cell lines
☒	☐	Palaeontology and archaeology
☐	☒	Animals and other organisms
☐	☒	Human research participants
☒	☐	Clinical data
☒	☐	Dual use research of concern

Methods

n/a	Involved in the study	
☒	☐	ChIP-seq
☐	☒	Flow cytometry
☒	☐	MRI-based neuroimaging

Antibodies

Antibodies used	ELISA: 0.25 μg/ml goat anti-human IgG(H+L)HRP (JACKSON, 109-035-003)<br>1 μg/ml H7N9 human IgG1 antibody HG1K (Sino Biologicals, Cat #HG1K) was used as negative control.<br>FACS: The cells were stained with FITC anti-human CD20 antibody (BioLegend, 302304), Brilliant Violet 421 anti-human CD27 antibody (BioLegend, 302824), PE/Cyanine7 anti-human IgM antibody (BioLegend, 314532), PE/Cyanine7 anti-human IgD antibody(BioLegend, 348210).<br>All human antibodies were expressed using Expi293F™ (Gibco, A14527) with codon-optimized cDNA and human IgG1 constant regions in house. The detailed sequence could be found in Supplementary material.
Validation	All antibodies were expressed using Expi293F™ with codon-optimized cDNA and human IgG1 constant regions. All antibodies' species and specificity to RBD were validated by ELISA. All antibodies neutralization ability was verified by VSV-based pseudotyped virus assays. Details for all SARS-CoV-2 antibodies evaluated in this study is included in Supplementary Table.<br>Goat anti-human lgG(H+L)HRP (JACKSON, 109-035-003): Based on immunoelectrophoresis and/or ELISA, the antibody reacts with whole molecule human lgG. It also reacts with the light chains of other human immunoglobulins. No antibody was detected against non-immunoglobulin serum proteins. The antibody may cross-react with immunoglobulins from other species.<br>FITC anti-human CD20 antibody was validated by successful staining and FC analysis according to the manufacturer's website https://www.biolegend.com/en-us/products/fitc-anti-human-cd20-antibody-558 and previous publication: Mishra A, et al. 2021. Cell 184(13):3394-3409.e20<br>Brilliant Violet 421 anti-human CD27 antibody was validated by successful staining and FC analysis according to the manufacturer's website https://www.biolegend.com/en-us/products/brilliant-violet-421-anti-human-cd27-antibody-7276 and previous publication Dugan HL, et al. 2021. Immunity. 54(6):1290-1303<br>PE/Cyanine7 anti-human lgM antibody was validated by successful staining and FC analysis according to the manufacturer's website https://www.biolegend.com/en-us/products/pe-cyanine7-anti-human-igm-antibody-12467 and previous publication: Shehata L, et al 2019. Nat Commun. 10:1126<br>PE/Cyanine7 anti-human lgD antibody was validated by successful staining and FC analysis according to the manufacturer's website https://www.biolegend.com/en-us/products/pe-cyanine7-anti-human-igd-antibody-6996 and previous publication: Ahmed R et al. 2019. Cell. 177(6):1583-1599.

Eukaryotic cell lines

Policy information about cell lines

Cell line source(s)	Monoclonal antibody expression: Expi293F™ (Gibco, A14527);<br>Yeast display: EBY100 (ATCC MYA-4941);<br>Pseudutyped virus neutralization assay: Huh-7 (JCRB 0403) ;<br>Authentic virus neutralizing assay:Vero(ATCC CCL-81);<br>293T(ATCC, CRL-3216);
Authentication	Expi293F™ (Gibco, A14527):Morphology(https://www.thermofisher.com/document-connect/document-connect.html?url=https://assets.thermofisher.com/TFS-Assets%2Fcertificate%2FFRK%2FCOA%2FCOA_100044202_275162_1.pdf);<br>EBY100 (ATCC MYA-4941):Whole-genome Sequencing(https://www.atcc.org/products/mya-4941);<br>Huh-7 (JCRB 0403):Morphology(https://cellbank.nibiohn.go.jp/~cellbank/en/search_res_det.cgi?ID=385);<br>Vero(ATCC CCL-81):Morphology(https://www.atcc.org/products/ccl-81#related-products);<br>293T(ATCC, CRL-3216):STR profiling(https://www.atcc.org/products/crl-3216)
Mycoplasma contamination	Not tested for mycoplasma contamination;
Commonly misidentified lines<br>(See ICLAC register)	No commonly misidentified cell lines were used in the study.

Animals and other organisms

Policy information about studies involving animals; ARRIVE guidelines recommended for reporting animal research

Laboratory animals	Female,6-8 weeks old BALB/c mice were used in this study
Wild animals	No wild animals were used.
Field-collected samples	No field-collected samples were used.
Ethics oversight	Animal experiments were carried out under study protocols approved by Institute of Biophysics, Chinese Academy of Sciences (SYXK2023300) and HFK Biologics (HFK-AP-20210930).

Note that full information on the approval of the study protocol must also be provided in the manuscript.

Human research participants

Policy information about studies involving human research participants

Population characteristics	We collected plasma samples from 50 convalescent individuals with BA.1 breakthrough infection, 22 long-term convalescent individuals with BA.1 breakthrough infection, 39 with BA.2 breakthrough infection, 36 with BA.5 breakthrough infection and 30 with BF.7 breakthrough infection, all of whom received three doses of CoronaVac before infection. Further, we investigated 26 individuals who had post-vaccination BA.1 breakthrough infection followed by BA.5/BF.7 reinfection, 19 with post-vaccination BA.2 breakthrough infection followed by BA.5/BF.7 reinfection, and 12 individuals with BA.1/BA.2 infection followed by BA.5/BF.7 reinfection, who had no history of vaccination. The gender, age, vaccination status, infection time, and sampling time were listed in Supplementary Table 1.
Recruitment	Patients were recruited on the basis of CoronaVac vaccination, post-vaccination BA.1, BA.2, BA.5 or BF.7 breakthrough infection, post-vaccination BA.1/BA.2 breakthrough infection followed by BA.5/BF.7 reinfection, and BA.1/BA.2 infection followed by BA.5/BF.7 reinfection, who had no history of vaccination. The exclusion criteria for the study included individuals with HIV or other debilitating diseases, as well as immunocompromised individuals.
Ethics oversight	Blood samples from vaccinated or unvaccinated individuals who had recovered from Omicron breakthrough infection or reinfection were obtained under study protocols approved by Beijing Ditan Hospital, Capital Medical University (Ethics committee archiving No. LL-2021-024-02) and the Tianjin Municipal Health Commission, and the Ethics Committee of Tianjin First Central Hospital (Ethics committee archiving No. 2022N045KY). All participants have provided written informed consent for the collection of information, storage and use of their clinical samples for research purposes, and publication of data generated from this study.

Note that full information on the approval of the study protocol must also be provided in the manuscript.

Flow Cytometry

Plots

Confirm that:

☒ The axis labels state the marker and fluorochrome used (e.g. CD4-FITC).

☒ The axis scales are clearly visible. Include numbers along axes only for bottom left plot of group (a 'group' is an analysis of identical markers).

☒ All plots are contour plots with outliers or pseudocolor plots.

☒ A numerical value for number of cells or percentage (with statistics) is provided.

Methodology

Sample preparation	Whole blood sample were diluted 1:1 with PBS+2% FBS (Gibco) and subjected to Ficoll (Cytiva) gradient centrifugation. Plasma was collected from upper layer. Cells were collected at the interface and further prepared by centrifugation, red blood cells lysis (Invitrogen eBioscience) and washing steps. Samples were stored in FBS (Gibco) with 10% DMSO (Sigma) in liquid nitrogen if not used for downstream process immediately. Cryopreserved PBMCs were thawed in PBS+2% FBS. CD19+ B cells were enriched from PBMCs using EasySep Human CD19 Positive Selection Kit II (STEMCELL, 17854). Following enrichment, 1x106 B cells in 100μl buffer were incubated with a panel of antibodies including 3μl FITC anti-human CD20 antibody (BioLegend, 302304), 3.5μl Brilliant Violet 421 anti-human CD27 antibody (BioLegend, 302824), 2μl PE/Cyanine7 anti-human IgD antibody (BioLegend, 348210) and 2μl PE/Cyanine7 anti-human IgM antibody (BioLegend, 314532). Additionally, fluorophore or oligonucleotide conjugated RBD were added. For FACS, 0.013μg of biotinylated BA.1 (Sino Biological, 40592-V49H7-B) or BA.2 (customized from Sino Biological) RBD protein conjugated with PE-streptavidin (BioLegend, 405204) and APC-streptavidin (BioLegend, 405207), and 0.013μg of WT biotinylated RBD protein (Sino Biological, 40592-V27H-B) conjugated with BV605-streptavidin (BioLegend, 405229) were added. For sequencing, BA.1 or BA.2 biotinylated RBD protein conjugated with TotalSeq™-C0971 Streptavidin (BioLegend, 405271) and TotalSeq™-C0972 Streptavidin (BioLegend, 405273), WT biotinylated RBD protein conjugated with TotalSeq™-C0973 Streptavidin (BioLegend, 405275) and TotalSeq™-C0974 Streptavidin (BioLegend, 405277) and biotinylated Ovalbumin (Sino Biological) conjugated with TotalSeq™-C0975 Streptavidin (BioLegend, 405279) were added. After incubation and washing steps, 5μl of 7-AAD (Invitrogen, 00-6993-50) was included for dead cell exclusion.
Instrument	Moflo Astrios EQ (BeckMan Coulter)
Software	Summit 6.0 (Beckman Coulter) for cell sorting; FlowJo 10.8 for data analysis.
Cell population abundance	BA.1 breakthrough infection (2m) : 7AAD-&CD20+/singletes=84.5%, CD27+&IgM-&IgD-/7AAD-&CD20+=23.6%, BA.1-RBD+/CD27+&IgM-&IgD-=0.28%, WT-RBD+/BA.1-RBD+=76.4 %<br>BA.2 breakthrough infection (2m) : 7AAD-&CD20+/singletes=90.9%, CD27+&IgM-&IgD-/7AAD-&CD20+=19.4%, BA.2-RBD+/CD27+&IgM-&IgD-=0.098%, WT-RBD+/BA.2-RBD+=70.6%<br><br>BA.1 breakthrough infection (8m) : 7AAD-&CD20+/singletes=79.3%, CD27+&IgM-&IgD-/7AAD-&CD20+=20.1%, BA.1-RBD+/CD27+&IgM-&IgD-=0.14%, WT-RBD+/BA.1-RBD+= 41.4%<br>BA.2 breakthrough infection (8m) : 7AAD-&CD20+/singletes=81.8%, CD27+&IgM-&IgD-/7AAD-&CD20+=23.9%, BA.2-RBD+/CD27+&IgM-&IgD-=0.19%, WT-RBD+/BA.2-RBD+= 39.9%

BA.1 breakthrough infection followed by BA.5/BF.7 breakthrough infection: 7AAD-&CD20+/singletes=83.1%, CD27+&IgM-&IgD-/7AAD-&CD20+=20.1%, BA.1-RBD+/CD27+&IgM-&IgD-=0.46%, WT-RBD+/BA.1-RBD+= 31.6%
BA.2 breakthrough infection followed by BA.5/BF.7 breakthrough infection: 7AAD-&CD20+/singletes=83.1%, CD27+&IgM-&IgD-/7AAD-&CD20+=23.2%, BA.2-RBD+/CD27+&IgM-&IgD-=0.18%, WT-RBD+/BA.2-RBD+=33.8%

BA.1 infection followed by BA.5/BF.7 breakthrough infection (without vaccination history): 7AAD-&CD20+/singletes=79.4%, CD27+&IgM-&IgD-/7AAD-&CD20+=34.5%, BA.1-RBD+/CD27+&IgM-&IgD-=0.11%, WT-RBD+/BA.1-RBD+= 14.6%
BA.2 infection followed by BA.5/BF.7 breakthrough infection (without vaccination history): 7AAD-&CD20+/singletes=83.4%, CD27+&IgM-&IgD-/7AAD-&CD20+=18.1%, BA.2-RBD+/CD27+&IgM-&IgD-=0.084%, WT-RBD+/BA.2-RBD+= 24.8%

Gating strategy

Cells negative for 7-AAD, IgM and IgD, but positive for CD20, CD27 and BA.1 RBD or BA.2 RBD were sorted, the gating strategy is provided in the Supplementary Information

☒ Tick this box to confirm that a figure exemplifying the gating strategy is provided in the Supplementary Information.
